# Characterization of subchronic lung and brain consequences caused by mouse-adapted SARS-CoV-2 and influenza A infection of C57BL6 mice

**DOI:** 10.3389/fimmu.2026.1755141

**Published:** 2026-02-25

**Authors:** Joshua Currey, Chenxiao Wang, Meredith G. Mayer, Yilin Chen, Ana Karina Nisperuza Vidal, Michaela J. Allen, Mst Shamima Khatun, Calder R. Ellsworth, Mohammad Islamuddin, Jefferson Evangelista, Skye M. Minor, Nadia Golden, Kevin J. Zwezdaryk, Nicholas J. Maness, Robert V. Blair, Jay K. Kolls, Derek A. Pociask, Tracy Fischer, Xuebin Qin

**Affiliations:** 1 Tulane National Biomedical Research Center, Covington, LA, United States; 2 Department of Microbiology and Immunology, Tulane University School of Medicine, New Orleans, LA, United States; 3 Center for Translational Research in Infection and Inflammation, Tulane University School of Medicine, New Orleans, LA, United States; 4 Department of Pulmonary Critical Care and Environmental Medicine, Tulane University School of Medicine, New Orleans, LA, United States

**Keywords:** mouse model, brain, influenza, long COVID, lung, MA30, PR8, SARS-CoV-2

## Abstract

**Introduction:**

SARS-CoV-2 and, to a lesser extent, influenza A can lead to long-term complications in the respiratory and nervous systems. However, the mechanisms driving post-viral sequelae remain poorly understood.

**Methods:**

To address this gap, we longitudinally characterized C57BL/6 mice infected with sublethal doses of mouse-adapted SARS-CoV-2 (MA30) or influenza A (PR8). Lung and brain tissues were analyzed at 14-, 21-, and 28-days post-infection (DPI) using histological analysis and bulk-RNA sequencing.

**Results:**

In the lungs, both infections caused prolonged inflammation and fibrosis. MA30-infected lungs showed persistent upregulation of inflammation, coagulation, complement, as well as fibrotic, and extracellular matrix (ECM) remodeling pathways at 21 DPI, alongside downregulation of epithelial junction and metabolic program pathways. In contrast, PR8-infected lungs exhibited a strong acute interferon response and chronic upregulation of basal epithelial markers (e.g., *Krt5*, *Krt14*), consistent with epithelial regeneration. Notably, only PR8-infected mice displayed KRT5+ progenitor cell migration into damaged lung regions, indicating divergence in repair mechanisms. Neither MA30-infected, nor PR8-infected mice had detectable brain infection. However, MA30 mice, but not PR8-infected mice exhibited an elevated frequency of microhemorrhages at early timepoints and marked neuroinflammation at all timepoints. Transcriptomic profiling of MA30-infected brains showed enrichment for up-regulation of ECM remodeling, vascular dysfunction, IL6-signaling pathways along with a virus-specific disruption of the hypothalamic–pituitary axis with MA30 infection not seen in PR8-infected brains. These included genes linked to neuroinflammation, sensory processing disruption, and microvascular injury, mirroring clinical features of Long COVID.

**Discussion:**

Together, these findings establish distinct tissue-specific trajectories of long-term pathology following SARS-CoV-2 and influenza infection and provide a foundation for dissecting the mechanisms of post-viral lung and brain disease.

## Introduction

Since its emergence in December 2019, SARS-CoV-2 (CoV2) has caused nearly 800 million infections and over 7 million deaths worldwide. In the aftermath of acute infection, a significant subset of individuals experience a prolonged illness termed Long COVID (LC). The U.S. Centers for Disease Control and Prevention (CDC) estimates that approximately 7.5% of adults develop LC after CoV2 infection, and nearly 18% of all infected individuals have experienced LC symptoms (1). LC encompasses a wide range of persistent symptoms, including fatigue, dyspnea, anosmia, ageusia, gastrointestinal dysfunction, and clotting abnormalities (2). Neurological and psychiatric manifestations such as anxiety, depression, brain fog, headaches, and sleep disturbances are also frequently reported (3–5). Although severe acute disease increases the risk of LC, individuals with mild or asymptomatic CoV2 infections can still develop lasting symptoms (6, 7). LC appears more prevalent in females despite males having higher acute disease severity and hospitalization rates (8). While influenza shares some features with COVID-19 disease, influenza does not have well documented long-term complications (9, 10).

The biological underpinnings of LC, especially in the lungs and brain, remain incompletely understood. Chronic inflammation and fibrosis have been implicated in long-term pulmonary sequelae (11–15), and Magnetic Resonance Imaging (MRI) and histological studies reveal brain changes, such as reduced gray matter thickness, structural damage, and loss of brain volume following CoV2 infection (16). While acute neuropathology such as neuroinflammation, microhemorrhages, and hypoxia has been documented in translationally relevant animal models, such as non-human primates by Rutkai et al. (17), few studies have examined the long-term molecular and histological alterations in the brain after CoV2 infection. Emerging evidence suggests LC may be driven by persistent inflammation, autoantibodies, unresolved tissue damage, or low-level viral persistence (18). However, how CoV2 triggers long-term neurological and respiratory symptoms remains poorly defined.

Like CoV2, influenza is known to cause acute lung injury, and recent work has suggested that some individuals experience persistent pulmonary symptoms post-infection (19–22). Studies in animal models have also shown that influenza can trigger prolonged immune activation and tissue remodeling, though these effects are less frequently studied than LC (23, 24). Investigating long term influenza is critical not only for understanding its own long-term impacts but also for identifying signatures unique to LC (25, 26). By comparing CoV2 and influenza head-to-head in animal models, we can begin to delineate which molecular and pathological changes are virus-specific versus shared consequences of respiratory infection (25, 26).

Various rodent models have been used to study the pathogenesis of CoV-2 and influenza infection. Syrian hamsters (Mesocricetus auratus) are also suitable models for CoV-2 and influenza infection (27–30). CoV-2-infected Syrian hamsters develop a moderate CoV-2 disease course, while CoV-2-infected Roborovski hamsters (Phodopus roborovskii) develop a severe disease course upon experimental infection (27, 28, 30). Murine models provide unique advantages for dissecting disease mechanisms due to their genetic tractability, cost-effectiveness, and the availability of molecular tools and transgenic lines. One prior study using MA10-infected BALB/c and aged C57BL/6 (B6) mice showed prolonged lung pathology and neuroinflammation in BALB/c animals but minimal changes in the B6 mice (31–33). Yet, B6 mice remain the preferred background for genetic and behavioral studies and many molecularly engineered lines are introduced to B6 background. Moreover, CoV2 infection induces fibrotic lung injury in both patients and animal models (34, 35) and our group previously demonstrated that K18-hACE2 mice exhibit long-term (21, 45 DPI) lung fibrosis after CoV2 infection (26, 36). Notably, basal KRT5+ progenitor cells, which contribute to lung repair after influenza (37–39), failed to migrate in these K18 mice following CoV2 infection (26). However, the K18 model is suboptimal for studying long-term brain pathology due to high viral loads in the brain not reflective of human disease (26, 40–42). Overexpression of hACE2, especially by cells not known to express ACE2 in humans, may also impact on non-physiological infection kinetics, particularly in both the lung and brain.

To better define post-viral effects unique to CoV2, there is a need for studies that directly compare it with other respiratory viruses such as influenza. To address this, we used a refined mouse-adapted CoV2 strain (MA30) and PR8 influenza virus to longitudinally assess infection outcomes in 3-month-old adult C57BL/6 mice. MA30 was serially passaged to enhance pathogenesis in non-aged mice and overcome limitations of the prior MA10 model (43). PR8 is a well-established mouse-adapted H1N1 influenza A strain that is well recognized to cause severe infections in mice (44, 45). Lung and brain tissues were evaluated at multiple timepoints post-infection (14, 21, and 28 DPI) using histopathology and transcriptomic analysis. This approach allowed us to map persistent inflammation, fibrosis, tissue repair dynamics, and neuroinflammatory signatures in a controlled setting. Our model enables a head-to-head comparison of CoV2 and influenza to distinguish shared and virus-specific features of long-term disease, providing a foundation for mechanistic insights into the development of post-acute viral outcomes.

## Materials and methods

### Ethics statement and mice information

All animal experiments were approved by the Institutional Animal Care and Use Committees (IACUC) at Tulane University and conducted under the animal use protocols: 1331, 1383, and 2288. 3-month-old age-matched control B6 mice were purchased from Jackson Laboratories for the studies. A total of 52 B6 mice (28 MA30-infected (male(n=14), female (n=14)), 18 PR8-infected (male (n=9), female (n=9)), and 6 non-infected (male (n=3), female (n=3)) were used in the Cov2 and influenzas’ study. These animals were housed in ventilated cages (Animal Care Systems, Inc., Littleton, CO) and maintained in a temperature- and humidity-controlled environment with a 12-hour light/dark cycle at Tulane University School of Medicine animal facility that is specific-pathogen-free. The B6 mice were intranasally infected with CoV-2 MA30 in a BSL-3 facility and intranasally infected with PR8 in a BSL-2 facility.

### MA30 and PR8 influenza infection and strain information

We obtained and used the same MA30 viral stock as published here (46). Mice were inoculated intranasally with MA30 (either 2.5x10^4^ or 5.0x10^4^ PFUs). For influenza viral infection, mice were infected with sublethal dose of H1N1 A/PR/8/34 (PR8, 50PFU) by oropharyngeal aspiration (23, 26, 45).

### Euthanasia and tissue collection

CO_2_ asphyxiation was used as a primary method of euthanasia with 30-70% flow rate of cage volume per minute of CO2 followed by cervical dislocation as a secondary method of euthanasia. Following euthanasia, tissues were collected by dissection of the lung and brain tissue. Tissue samples from each organ of interest were stored separately in Trizol Reagent (Invitrogen Cat#:15596018) or buffered 10% zinc-formalin fixative (Anatech Cat#:SKU171) and fixed for virus inactivation prior to their removal from BSL2 or BSL3 laboratories. Tissues stored in buffered zinc-formalin fixative were processed for paraffin embedding.

### Body weight measurements

Body weight measurements were taken before infection and daily after infection with MA30 for 28 days post-infection in the ABSL3 laboratory and for PR8 infected mice for 28 DPI after infection in ABSL2 laboratory then recorded in grams.

### RNA isolation, quantification, and purity testing

Tissue samples for MA30 and PR8 infection were preserved in 1mL Trizol (15596026; Invitrogen) and extracted with a RNeasy Mini Kit (Cat. No.74104; QIAGEN) according to the manufactures’ instructions. Nanodrop was used to test the concentration and purity of the RNA prior to qPCR and bulk RNAseq.

### Plaque assay

Lung samples were weighed and homogenized in 1.5 mL serum-free DMEM (Thermo Scientific, USA). Plaque assays were performed in 12-well plates using confluent Vero-TMPRSS2 cells (JCRB Cell Bank, Japan), with duplicate samples diluted from neat to 1×10^−4^. After incubation, the inocula were removed and replaced with 1 mL overlay (1× MEM, 2% FBS, 1.2% Avicel), followed by 48 hr incubation at 37 °C/5% CO_2_. Wells were washed with PBS, fixed with 10% formalin for 1 hr at room temperature, then stained with 0.1% crystal violet. Plaques were counted, and PFU/mL was calculated: (plaques × dilution factor) ÷ inoculum volume.

### Subgenomic nucleocapsid gene RNA copy number detection

Subgenomic N RNA copy number was determined as described in the previous publication for both MA30 infected lung and brain tissue using qPCR. M1 qRT-PCR was used to determine viral titter of PR8 after infection (47). Results were obtained and quantified using the ABI QuantStudio 6 system following the same protocol as used for MA30 infection in (46) and PR8 infection in (47).

### Lung and brain histological and IHC staining

Lung and brain sections from MA30- or PR8-infected mice were deparaffinized and assessed by hematoxylin and eosin (H&E), trichrome (lung only), or immunohistochemistry (IHC) using chromogen- or fluorescent-labeled antibodies. Lung and brain slides were digitally scanned and analyzed using HALO software (Indica Labs, 2024) (26). Inflammatory areas in lung were quantified based on cellular infiltration. Brain microhemorrhages were manually annotated and counted from H&E-stained sections. Microhemorrhage frequency was normalized to total tissue area, and average bleed area per mouse was calculated.

For lung IHC, CoV2 antigen was detected using a guinea pig polyclonal anti-SARS-CoV-2 antibody (BEI NR-10361) (1:1000) with an AF488-conjugated goat anti-guinea pig secondary (Invitrogen A-11073) (1:1000) (26). Krt5+ cells were labeled using a chicken polyclonal anti-Krt5 antibody (BioLegend #905901) (1:1000) and goat anti-chicken AF488 secondary (Invitrogen A11039) (1:1000) (26). Viral signal was quantified per tissue area using Highplex FL v4.2.14 (HALO v3.6).

Brain IHC was performed using anti-Iba1 (Wako 019-19741) and anti-GFAP (Abcam ab7260) primaries, followed by a horse anti-rabbit secondary (Vector Labs BA-1100) and Vector Red substrate. Sections were counterstained with Mayer’s hematoxylin. Manual review was performed to verify all brain annotations.

### Bulk RNA-sequencing analysis

Final cDNA libraries containing TruSeq RNA CD indexes (Illumina, 20019792) were quantitated using a Qubit dsDNA HS assay kit (Thermo Fisher Scientific, Q32854). The quality of the libraries was determined by running each on an Agilent TapeStation 4150 using an Agilent D1000 ScreenTape (Agilent: 5067-5582). Smear analysis was performed using Agilent TapeStation Software (Version 4.1.1) with a range of 200-600bp to determine the average size of each library. Size and concentration were then used to calculate the molarity of each library.

Libraries were pooled at a final concentration of 750pM with a spike-in of 2% PhiX control library v3 (Illumina, FC-110-3001). A mixture of pooled libraries was loaded on an Illumina NextSeq P1 (300) reagent cartridge (Illumina, 20050264). Paired-end and dual indexing sequence, 150x8x8x150, was performed on NextSeq2000, yielding approximately 20M paired-end reads per sample. Fastq files generated by Illumina BaseSpace DRAGEN Analysis Software (Version 1.2.1) were applied for further data analyses. Raw reads were assessed using FastQC before alignment to mm10 by STAR. Transcript assembly and abundance estimation were performed with Cufflinks. For gene-level differential expression, quantified reads were analyzed with DESeq2. Genes with adjusted p-value < 0.05 (Benjamini–Hochberg method) and |log2 fold change| > 1 were considered significantly differentially expressed. Volcano plots were generated using the Enhanced Volcano package (v1.16.0) to visualize gene-level changes. Gene set enrichment analysis (GSEA) was conducted using clusterProfiler (v4.14.6). Mouse-specific gene sets from the C2 (curated) and C5 (Gene Ontology) categories of the MSigDB database were retrieved using msigdbr (v7.5.1). Gene sets with adjusted p-value < 0.05 were considered significantly enriched, and results were visualized using enrichplot, ggplot2, and base R graphics.

### Statistics

Data are presented as mean ± standard error of the mean (SEM). For comparisons between two groups, unpaired Student’s *t*-tests were used. For experiments involving multiple groups and time points, two-way ANOVA, mixed model with Tukey’s multiple comparisons. A *P* value < 0.05 was considered statistically significant. All analyses were performed using GraphPad Prism version 10.1.

## Results

### B6 mice infected with a sublethal dose of MA30 and PR8 lost body weight and did not have detectable viral load in the lungs at 14–28 DPI

We investigated the ability of infection with MA30 to elicit subchronic consequences after acute infection in 3-month-old male and female B6 mice (n=28), a normal age widely used for experimental investigation of the pathogenesis in mice models of human disease. MA30 infection was administered at either 2.5x10^4 and 5.0x10^4 TCID50, sublethal doses to induce maximal pathological effect while still having surviving animals to test subchronic pathological effects. These dosages were slightly higher than the sublethal dose we determined in our previous study (46) (**Supplementary Figures 1A, B**, **2A**). To explore the distinct subchronic consequences of MA30 infection, we compared them with 3-month-old and sex matched B6 mice infected with a sublethal dose of influenza PR8 (26) (**Supplementary Figure 2B**). We euthanized at 4 time points for MA30 (5-7, 14-16, 21, and 28 DPI) and PR8-infected mice (14, 21, and 28 DPI) to thoroughly examine disease progression as well as if subchronic impacts could be observed for up to 28 DPI. While we used 2 different doses (2.5x10^4 or 5.0x10^4 TCID50) to infect our mice with MA30, we did not see any notable differences in the body weight and survival rate between the two groups (**Supplementary Figures 1A, B**). Thus, we decided to group these two dosages together for further analysis.

**Figure 1:**
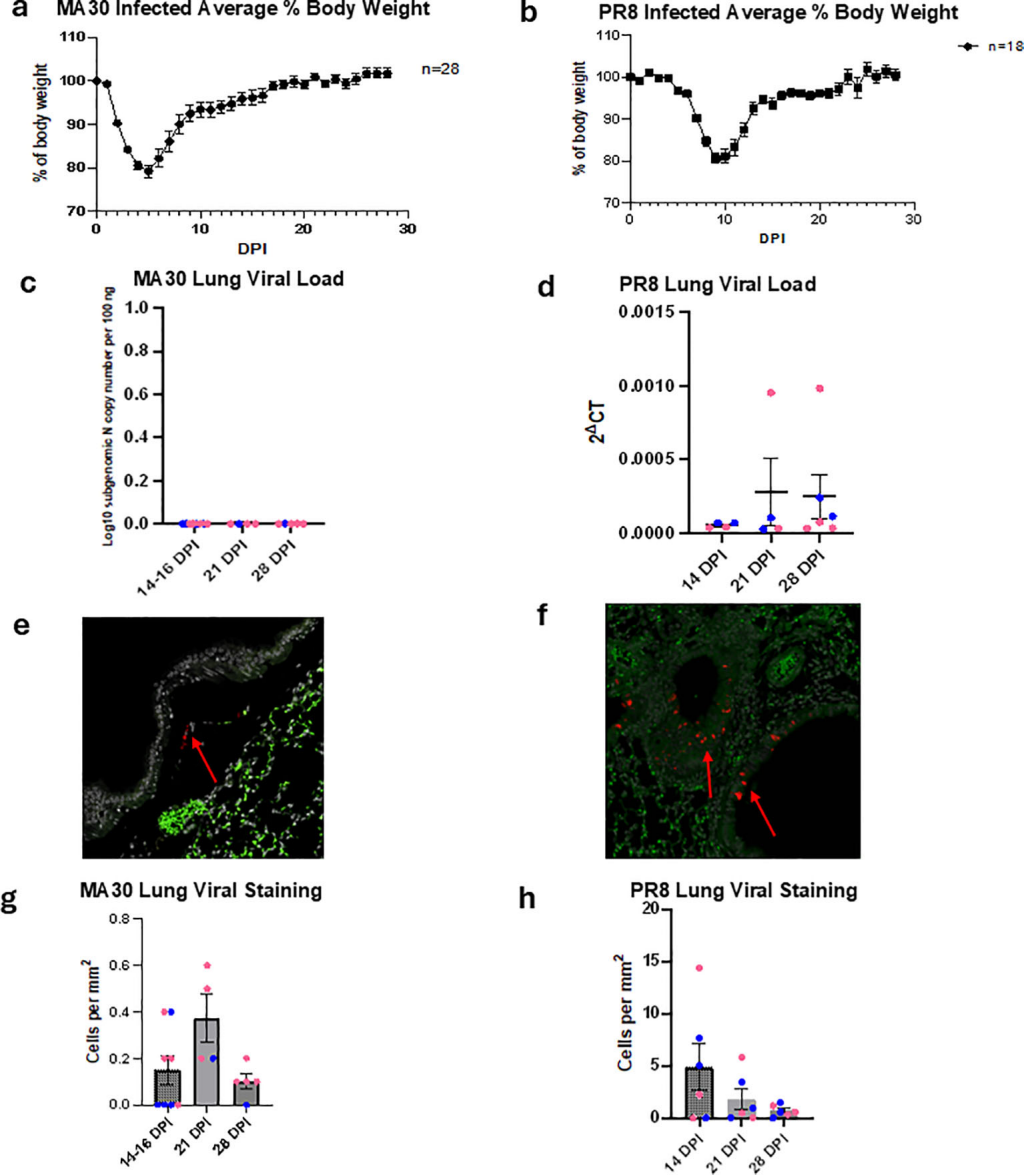
Sublethal infection with SARS-CoV-2 MA30 or influenza A PR8 induces acute weight loss and some viral antigen persistence in the lungs of C57BL/6 mice. **(A, B)** Mice were infected intranasally with sublethal doses of either MA30 or PR8 and monitored for up to 28 days post-infection (DPI). Both groups showed approximately 20% body weight loss during acute infection (5–9 DPI), with recovery to 90–95% of baseline weight by 28 DPI. **(C, D)** qPCR indicated no viral load at 14–28 DPI. **(E–H)** Lung IHC staining showed some low-level viral antigen persistence for up to 28 DPI. Each dot represents an individual mouse (blue = male, pink = female); sample sizes were n = 4–6 per group, and error bars indicate SEM.

Mice infected with MA30 at 5–7 DPI and PR8 at 8–9 DPI had lost around 20% total body weight (**Figures 1A, B**). Mice that survived for up to 28 DPI with MA30 or PR8 returned to nearly 90-95% total body weight (**Figures 1A, B**). MA30 had a 40% and PR8 had a 0% mortality rate (**Supplementary Figure 2B**). Equal amounts of males and females were used in our study to determine if sex-based differences were observed in either MA30- or PR8-induced disease. In PR8-infection we did not observe any difference in body weight loss or survival between males and females(**Supplementary Figure 3a**). In contrast in MA30 infection, male mice exhibited significantly greater body weight loss and a higher mortality than female mice(**Supplementary Figures 3b, c**).

We assessed viral loads of MA30 and PR8 in tissue using subgenomic qPCR and IHC. For IHC, CoV2 antigent detection in MA30-infected lungs was performed using anti-SARS polyclonal antibodies, while influenza A virus was detected using an anti-nucleoprotein antibody (**Figures 1C–G**). Plaque assay was employed to validate findings from our qPCR and IHC studies to test for replicating MA30 virus at 5-7, 14–16 and 21 DPI (**Supplementary Figure 4**). Replicating MA30 virus was only detected by plaque assay in lungs collected at 5–7 DPI but not at any other time points (**Supplementary Figure 4**). These results further confirm our previous findings of active viral replication in MA30 infected mice at 5–7 DPI (46, 48, 49). At later time points (14-16, 21, and 28 DPI), IHC detected limited anti-SARS (MA30) viral staining (**Figures 1E, G**) and anti-nucleoprotein (PR8) staining (**Figures 1F, H**).

### Persistent lung inflammation and chronic collagen deposition in MA30- and PR8-infected B6 mice

**Figure 2:**
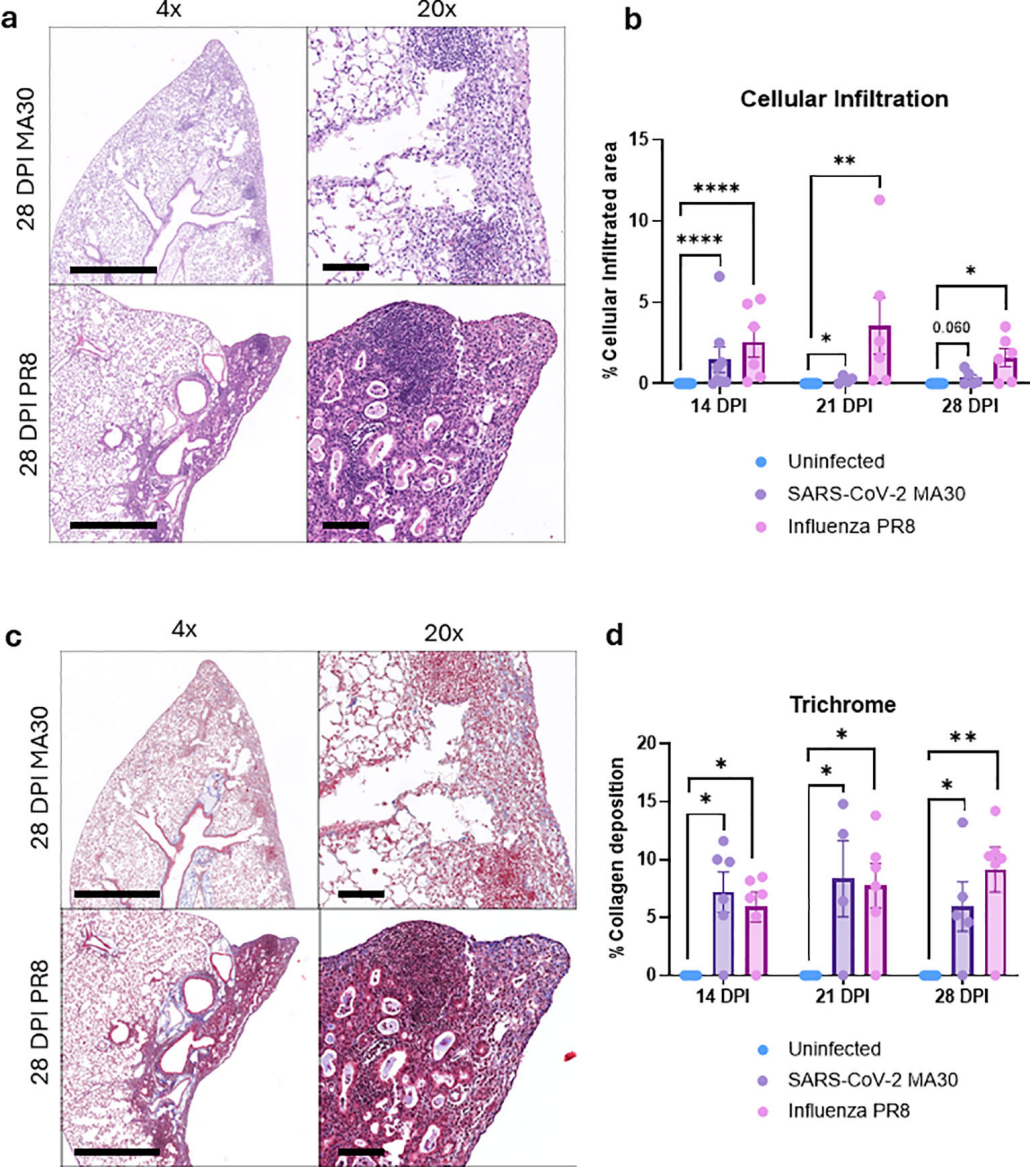
MA30 and PR8 infection leads to persistent pulmonary inflammation and unresolved collagen deposition in the lungs of C57BL/6 mice. Lung tissue was collected at 14-, 21-, and 28-days post-infection (DPI) from mice infected with SARS-CoV-2 MA30 or influenza A PR8. **(A, B)** Hematoxylin and eosin (H&E) staining revealed widespread and persistent immune cell infiltration in both MA30- and PR8-infected lungs through 28 DPI. **(C, D)** Masson’s trichrome staining showed progressive and unresolved collagen deposition, consistent with ongoing fibrotic remodeling in the lungs at 28 DPI in both PR8 and MA30. *P < 0.05 and **P<0.01 were compared between the two groups by Two-sided T-test. 28 DPI MA30: MA30-infected lungs at 28 days DPI, and 28 DPI PR8: PR8-infected lungs at 28 DPI. Bar (4X) = 1mm; Bar (20X) = 100um. ****p < 0.0001.

To assess long-term pulmonary pathology following viral infection, we quantified inflammatory cell infiltration and collagen deposition in the lungs of B6 mice infected with either MA30 or PR8. Histological analysis of hematoxylin and eosin (H&E)-stained lung sections revealed widespread immune cell infiltration in both MA30- and PR8-infected animals at 14, 21, and 28 days post-infection (DPI), indicating persistent inflammation beyond the acute phase (**Figures 2A, B**). Quantification of infiltrated areas confirmed that inflammation remained unresolved for at least four weeks in both infection models. Given that Long COVID has been associated with fibrotic lung remodeling, we next evaluated collagen accumulation using Masson’s trichrome staining. Strikingly, both MA30 and PR8 groups exhibited ongoing collagen deposition and fibrotic changes in the pulmonary interstitium at 14, 21, and 28 DPI (**Figures 2C, D**), with no evidence of resolution over time. These findings suggest that sublethal respiratory viral infections with either CoV2 or influenza can lead to chronic lung remodeling characterized by sustained inflammation and fibrosis, with potential implications for post-viral respiratory dysfunction.

### Impaired Krt5+ progenitor cell migration post MA30 but not PR8 infection

**Figure 3:**
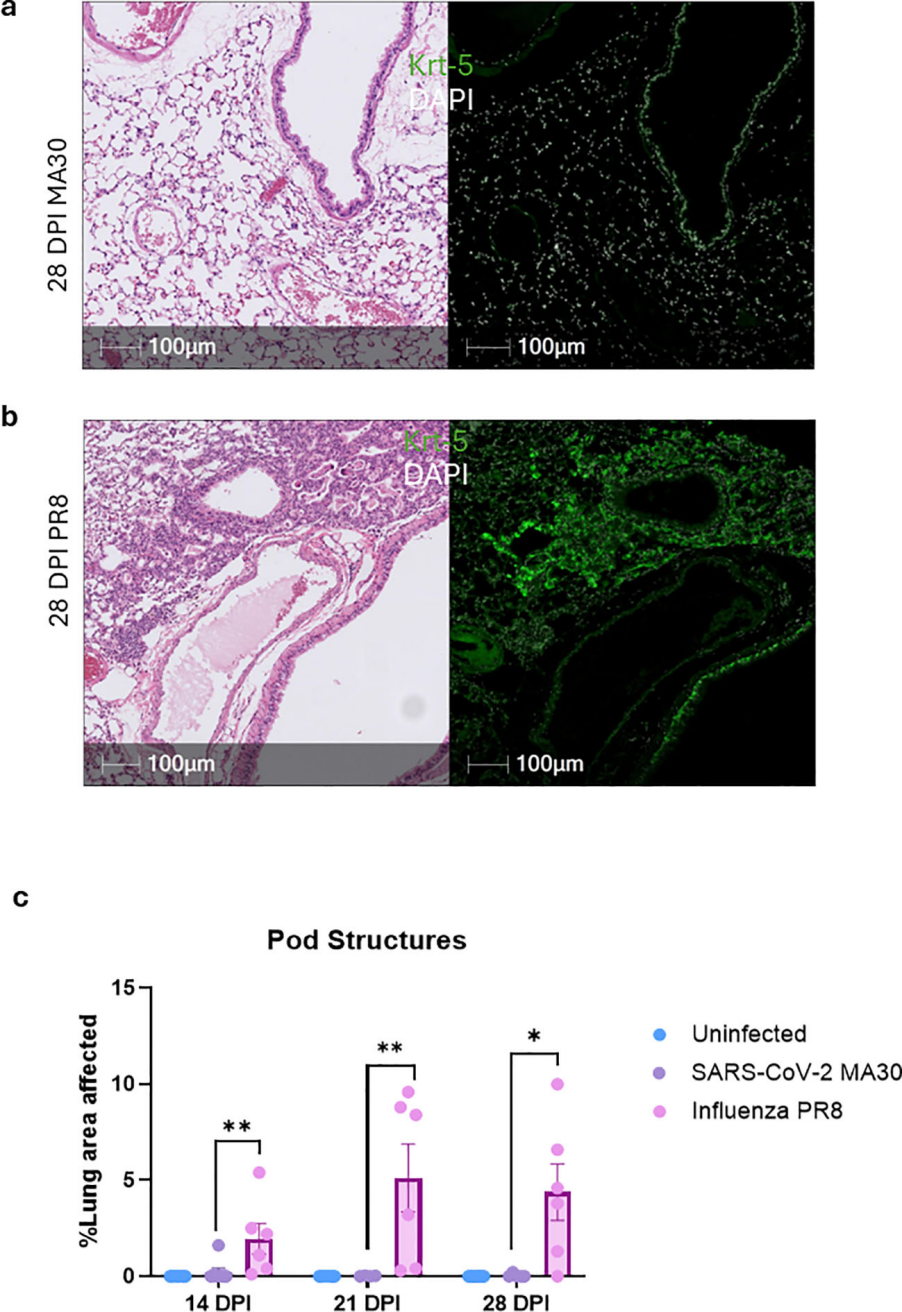
KRT5+ progenitor cell migration and pod formation are impaired following MA30 infection but preserved after PR8 infection. Lung sections from SARS-CoV-2 MA30- or PR8-infected C57BL/6 mice were stained by immunohistochemistry (IHC) for KRT5 at 14,21, and 28 days post-infection (DPI) to assess epithelial repair responses. **(A)** In PR8-infected lungs, KRT5+ cells were observed forming distinct pod-like structures within areas of consolidation, consistent with regenerative basal cell migration and proliferation. **(B)** In contrast, MA30-infected lungs lacked KRT5+ pod formation in similar regions, indicating a failure to recruit or expand progenitor cells at sites of injury. **(A, B)** Serial H&E and KRT5-stained sections confirmed that pod structures colocalized with consolidated regions in PR8 but were absent in MA30-infected lungs. **(C)** Quantification by computer software confirmed visual findings of pod-structure only forming in PR8 infected but not in MA30 infected lung tissue. *P < 0.05 and **P<0.01 were compared between the two groups by Two-sided T-test. 28 DPI MA30: MA30-infected lungs at 28 days DPI, and 28 DPI PR8: PR8-infected lungs at 28 DPI.

KRT5 progenitor cells are an essential population that helps repopulate pneumocytes after influenza infection, particularly within consolidated regions of the lung—areas where normal air-filled spaces are lost due to fibrosis, edema, inflammatory infiltrates, or epithelial remodeling (50). Recently we demonstrated that CoV2 infected K18 mice fail to elicit KRT5 cell migratation and do not form pod structures in the lung at 7, 14, or 21 DPI (26). This is different than PR8-infected mice which form robust pod structures and elicit migration of the KRT5 progenitor cells to the consolidated region at 7, 14, and 21 DPI (26). To further evaluate these findings in a different CoV2 model, we utilized MA30-infected and PR8-infected B6 mice. We have documented that in the case of PR8- but not MA30- infected mice, there is significant migration and proliferation of KRT5+ cells to consolidated lung regions at 28 DPI (**Figures 3A–C**) and other time points such as 14 DPI or 21 DPI (**Supplementary Figures 5A, B**) as seen in our IHC for KRT5 as well as in the consolidated regions in H&E staining. These results demonstrated that KRT5 pod structures overlap with consolidated regions in PR8 infected mice lungs but not in MA30. In summary, MA30 and PR8 sublethal infection of B6 mice causes persistent inflammation and fibrosis in the lung but KRT5 pod structure formation is only seen in PR8 but not MA30 infection.

### Lung transcriptomic changes in MA30- and PR8-infected B6 mice

To comprehensively investigate the dynamic transcriptomic changes in the lungs in response to Cov2 and influenza infections, we studied them at acute and subchronic phases. We conducted bulk RNA analysis of the lungs collected from MA30- and PR8 infected B6 mice at acute (7, and 8–9 DPI for MA30 and PR8 infection, respectively) and subchronic phase (21 DPI for both MA30 and PR8 infection). For acute phase transcriptomics study, we used the lung samples collected from MA30-infected mice at 7 DPI. Those mice were euthanized because they met humane endpoint criteria. In contrast, for influenza-infected mice, the infection dose of 50 PFU of PR8 was not high enough to induce the severity that met humane endpoints. To match the severity and the disease progression induced by MA30 infection, we used lung samples collected from PR8-infected mice at 8–9 DPI (n=4) infected with a lethal dose (83 PFU) from a different cohort.

At the subchronic phase (or at 21 DPI) of the infections, the MA30 infection upregulated complement genes (*C1qa, C1qb, C1qc, C6*, and *C3ar1*) (**Supplementary Table 1**), coagulation factors or related genes (*Fga*, *Fgg*, and *Alb*), and pro-inflammatory markers such as *Cxcl10* and *Tnf* in the lungs as compared to non-infected mice(**Supplementary Table 1**; **Supplementary Figure 6A**). PR8 infection upregulated interferon-responsive genes (*Ifit1*, *Stat1*, *Ifng*, *Irf7*) and markers of epithelial regeneration, including Krt5, Krt15 and Krt4, and pro-inflammatory markers such as *Cxcl9, Cxcl10, Ccl3, Ccl5, Ccl19, Cxcr3*, and *Tnf* in the lungs as compared to non-PR8 infection (**Supplementary Figure 6B**).

Pathway analyses in MA30-infected lungs at 21 DPI demonstrated enrichment of complement and coagulation pathways, ECM receptor interaction, Chemokine receptors bind chemokines, cytokine and cytokine receptor interaction, and PPAR signaling pathway (**Supplementary Figure 7A**). In contrast, the tight junction signaling pathway was downregulated, suggesting ongoing epithelial barrier dysfunction (**Supplementary Figure 7B**). Meanwhile, influenza-infected lungs at 21 DPI maintained enrichment for up-regulation of cytokine-cytokine receptor interaction, IFN gamma and beta pathways, ECM regulation, and Toll-like receptor signaling pathway (**Supplementary Figure 8A**) and down-regulation of muscle contraction, and PPAR signaling pathway (**Supplementary Figure 8A**).

**Figure 4:**
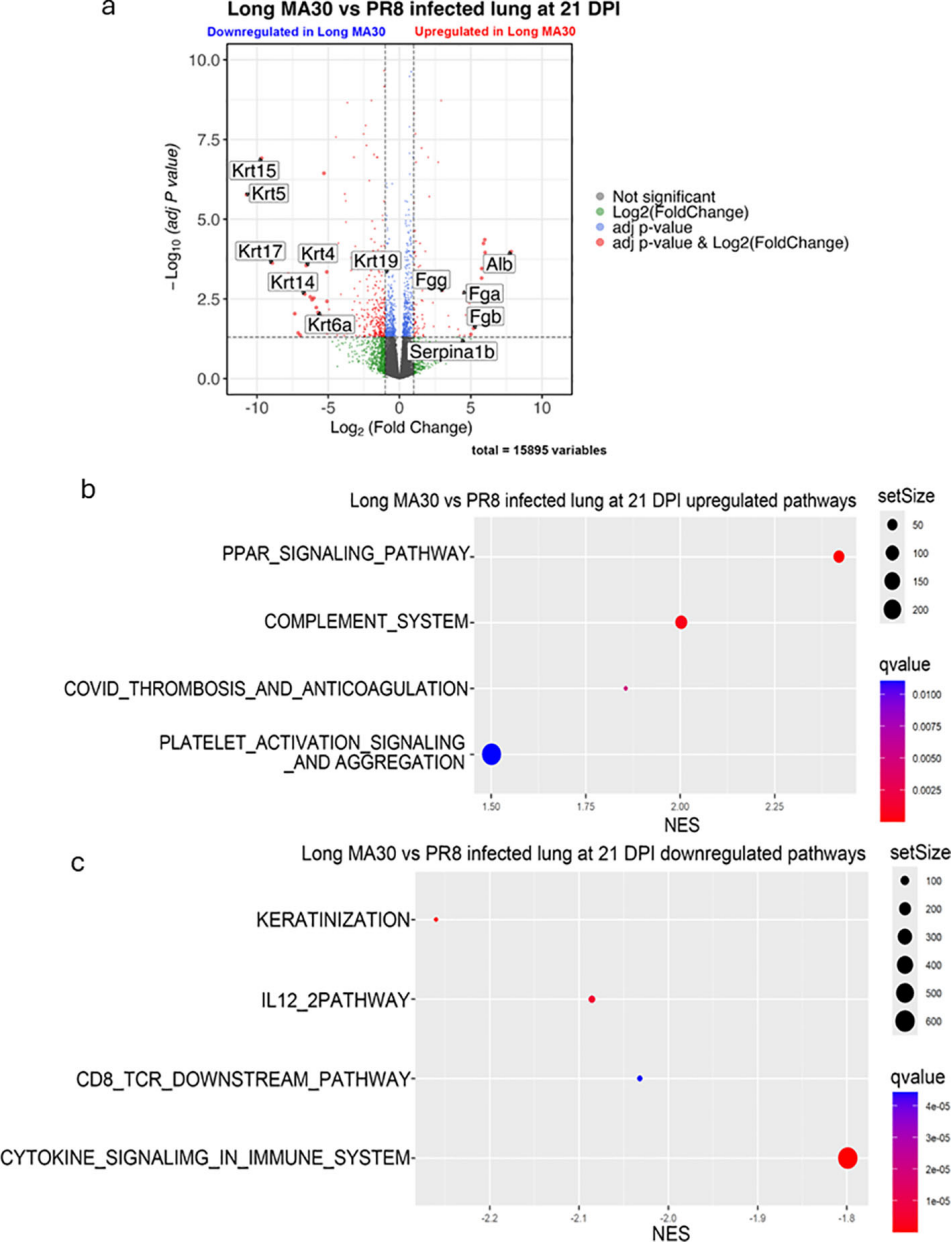
Long-term MA30 and PR8 infection induce distinct lung transcriptional profiles at 21 DPI. **(A)** Volcano plot shows differentially expressed genes in lungs from MA30- (n=6) and PR8 (n=4) -infected mice. **(B)** Pathway enrichment of upregulated genes reveals MA30-specific activation of complement, thrombosis, platelet activation and aggregation, and PPAR signaling pathways. **(C)** In contrast, PR8 lungs are enriched for keratinization, IL12 signaling, CD8 TCR downstream pathway, and cytokine signaling in immune system pathways, indicating more effective regeneration. These data highlight divergent immune and repair dynamics in long COVID versus long flu lungs.

Further, we conducted side-by-side comparison of the MA30-infected with PR8-infected pulmonary transcriptomes and revealed distinct long-term lung responses to those two viral infections. Subchronic COVID lungs had significantly increased expression of coagulation factors and related genes including *Fgg* (fibrinogen gamma chain), *Fga* (fibrinogen alpha chain), *Fgb* (fibrinogen beta chain), *Serpina3k*, *Serpina1b*, and *Serpina1c* (**Supplementary Table 2**), and reduced expression of Keratin family genes such as *Krt5*, *17*, *15*, *14*, *4*, *6A*, and *19* (**Figure 4A**) as compared to PR8-infected lungs. Pathway comparison revealed that subchronic MA30-infected lungs had the upregulation of the pathways for PPAR signaling pathway, complement system, COVID-19 thrombosis and anticogulation, and platelet activation signaling and aggregation (**Figure 4B**) as compared to subchronic PR8-infected lungs (**Figure 4C**). Subchronic MA30-infected lung had the down-regulation of the pathways for keratinization, IL-12–2 pathways, CD8 TCR downstream pathway, and cytokine signaling in immune system as compared to subchronic PR8-infection lungs (**Figure 4C**).

To show the distinct transcriptomic changes between CoV2 and influenza infections, we also conducted pairwise comparison of the MA30 with PR8-induced pulmonary transcriptomes at the acute phase of the infections. MA30 infection triggered early upregulation of complement components (*C7* and *Cfi*), coagulation regulators (Serpina3k,Serpina9, and *Alb*) (**Supplementary Table 3**), and Fibriogen family genes such as *Fgb*, *Fgl1* (Fibriogen-like protein1) as compared with PR8-infection (**Supplementary Table 3**; **Supplementary Figure 9**). In contrast, MA30 infection down-regulated expression of keratin family genes (*Krt6a*, *Krt5*, *Krt17*, *Krt15*, *Krt20*, and *Krt 14*), *Ifng*, complement genes (*C1qa*, *C1qb*, *C3a1*, *C5ar1*, *C5ar2*, *Cfp*, *Cfb*, *C2*, *C4a*) in the lung as compared with PR8-infection (**Supplementary Table 3**; **Supplementary Figure 9**). Acute MA30-infected lungs in the mice had enrichment for multiple pathways including up-regulation of collagen chain trimerization, collagen degradation, degradation of the extracellular matrix, COVID thromobosis and anticoagulation cascades, complement and coagulation cascades, and down-regulation of keratinization, type II interferon signaling, IFNG, Interferon signaling, Toll receptor and MHC class II antigen presentation in the lung as compared to acute PR8-infected lung (**Supplementary Figures 10A, B**). Collectively, we found both infections induce inflammatory pathways and inflammation related genes upregulation. MA30 infection induced acute and long term effects in the lung mainly with upregulation of complement, coagulation and thrombosis pathways, and downregulation of keratinization as compared to PR8-induced effects in the lung, while acute PR8 infection induced more interferon and IFN-gamma signaling than MA30-infection in lung.

### Increased microhemorrhage size and frequency in acute infection

**Figure 5:**
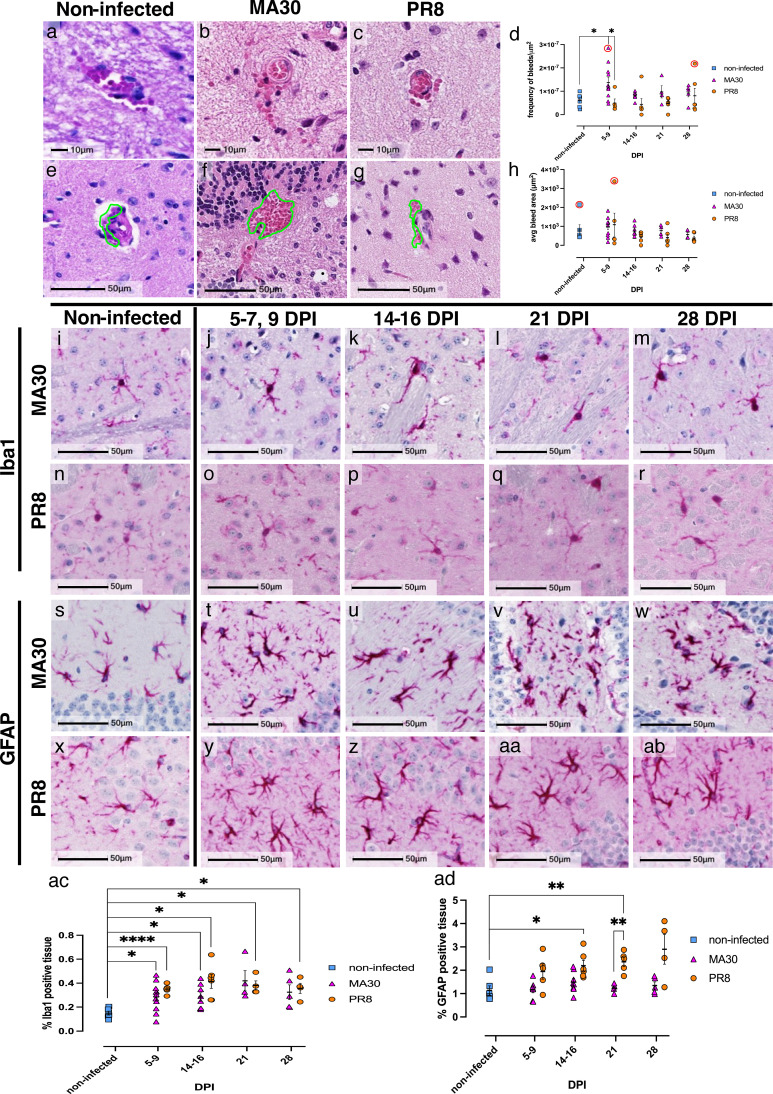
Microhemorrhage and inflammatory glial morphology with MA30 infection. H&E exposed more frequent brain microhemorrhages normalized to tissue area **(D)**, in 5–7 DPI MA30 infection **(B, F)**, as compared to non-infected (**A, E**, p = 0.0197), and 8–9 DPI PR8-infected mice (**C, G**, p = 0.0195). Statistical outliers were identified using the ROUT method (Q = 1%) and are indicated by red circles. The statistical significance at 5–9 DPI between MA30- and PR8-infected and non-infected animals remained unchanged after removal of outliers. Average bleed area was determined by manual annotation of red blood cells outside the vasculature via HALO imaging software **(H)**, represented as green outlines **(E–G)**. Although larger bleed areas were observed at 5–7 DPI in MA30-infected mice, compared to non-infected and 8–9 DPI PR8-infected animals, the differences did not reach statistical significance **(H)**. Removal of two identified outliers (red circles) revealed a significant increase in average bleed area of 5–7 DPI MA30-infected mice, as compared to non-infected controls (**H**, p = 0.0469). Expression of Iba1 and GFAP by IHC demonstrated inflammatory microglia and astrocyte morphology in brain from MA30-infected mice, respectively. In the context of MA30 infection, microglia **(J–M)** have more retracted cell processes, larger soma, and darker Iba1 intensity, compared to non-infected controls **(I, N)** and PR8-infected mice **(O–R)**. Iba1 positive area increased after both MA30 and PR8 infection compared to non-infected controls at a statistically significant level [non-infected and 5–7 DPI MA30-infected (adjP = 0.0105); non-infected and 9 DPI PR8-infected (adjP <0.0001); non-infected and 14–16 DPI MA30-infected (adjP = 0.0167); non-infected and 14–16 DPI PR8-infected (adjP = 0.0118); non-infected and 21 DPI PR8-infected (adjP = 0.0106); and non-infected and 28 DPI PR8-infected (adjP = 0.0244)] **(ac)**. Tissue sections without comparable hippocampus and cortex regions were not included in this analysis. Two animals were removed from both 21 DPI and 28 DPI PR8 infection timepoints. Representative images of hippocampal astrocytes in non-infected animals displayed highly ramified processes with reduced soma **(S, X)**, whereas astrocytes in MA30 post-infected animals **(T–W)** had more intense GFAP expression, thickened processes, and larger cell bodies. Greater GFAP expression and moderate thickening of astrocyte processes with enlarged soma were observed in PR8-infected mice **(y-ab)**, without retracted processes. Greater GFAP positive area was observed in PR8-infected animals compared to non-infected controls [14–16 DPI (adjP = 0.0144) and 21 DPI (adjP = 0.0044)] and MA30 at 21 DPI (adjP = 0.0058) **(ad)**. Tissues that did not include comparable hippocampus and cortex regions were excluded from analysis including three animals from MA30 5–7 DPI, one from PR8–21 DPI, and two from PR8–28 DPI.

Although virus was not detected in the brain of MA30-infected and PR8-infected mice (**Supplementary Figure 11**), evident neuropathological changes were observed only in MA30-infected mice (**Figure 5**; **Supplementary Figure 12**). Examination of H&E-stained brain tissue revealed an increase in microhemorrhage frequency in acute MA30 infection (5–7 DPI; **Figures 5B, F**), as compared to non-infected (**Figures 5A, E, D**; adjP = 0.0197) and PR8-infected animals at 8–9 DPI (**Figures 5C, G, D**: adjP = 0. 0195). This remained statistically significant when MA30 5–9 DPI outlier, as defined by the ROUT test (Q = 1%), was removed. At later timepoints, however, microhemorrhorage frequency in MA30-infected animals was similar to non-infected controls. PR8-infected animals had fewer microhemorrhages compared to MA30 infection throughout the later timepoints, although this did not reach statistical significance.

In addition to the increase in microhemorrhage frequency during acute infection, an observable increase in microhemorrhage size is seen in the context of MA30 but not PR8 infection (**Figures 5E–G**, green outline). The annotated bleed area was normalized to the bleed count for each tissue section. This demonstrated an increase in the average bleed area at 5–7 DPI compared to naïve controls (**Figure 5H**). Removal of statistical outliers, identified by ROUT (Q = 1%), did not change the statistical significance between the MA30- and PR8-infected groups at 5–9 DPI (**Figure 5H**). However, removal of two statistical outliers revealed significantly larger microhemorrhages in MA30-infected animals at 5–7 DPI, as compared to non-infected controls (p=0.0469; **Figure 5H**).

### Inflammatory glial morphology persists after acute infection

Inflammation in the brain was assessed by IHC using antibodies to identify microglia (ionized calcium-binding adapter molecule 1, Iba-1) and astrocyte (glial fibrillary acidic protein, GFAP) morphology. Evidence of infiltrating leukocytes was not observed in H&E stained brain tissue, however, Iba-1 revealed altered microglial morphology suggestive of a pro-inflammatory activated state, with retracted, thickened processes throughout all timepoints with MA30 infection (**Figures 5J–M**). Microglial polarity was often observed, which may indicate cell migration. Compared to brain from MA30-infected animals, microglia in brain of non-infected mice have a more ramified morphology that is consistent with a homeostatic, surveying phenotype (**Figures 5I, N**, ac; 5–9 DPI adjP = 0.0105; 14–16 DPI adjP = 0.0167). Similarly, PR8-infected animals’ microglia structure was largely suggestive of homeostatic phenotypes but have increased process thickness and enlarged cell bodies, encompassing more tissue area as Iba1 positive compared to non-infected controls (**Figures 5O–R**, ac; 5–9 DPI adjP < 0.0001; 14–16 DPI adjP = 0.0118; 21 DPI adjP = 0.0106; and 28 DPI adjP = 0.0244). Critically, nodular lesions were observed in the brains of MA30-infected animals but were not seen PR8-infected or naïve animals (**Supplementary Figure 12**).

Consistent with neuroinflammatory changes in the brain, astrocytes also exhibited signs of activation, including upregulation of GFAP, loss of cellular domain, retracted processes and enlarged cell bodies in both MA30 and PR8 infection, as compared to non-infected animals (compare **Figures 5t–w**, **5y–ab** to **5s, x**), but overall, GFAP positive area was only significantly increased in PR8-infected animals (**Figures 5A, D**, 14–16 DPI PR8 and non-infected adjP = 0.0144, 21 DPI PR8 and non-infected adjP = 0.0044). Morphological changes indicating an activated state were more severe in the context of MA30 infection, as compared to animals infected with PR8 (compare **Figures 5T–W** TO **5Y–AB**) but activated GFAP positive cell area after PR8 infection was greater than in MA30-infected mice (**Figures 5A, D**, 21 DPI adjP = 0.0058). Together, although neither MA30-infected, nor PR8-infected mice had detectable brain infection, MA30 mice, but not PR8-infected mice exhibited an elevated frequency of microhemorrhages at early timepoints and marked neuroinflammation at all timepoints.

### Brain transcriptional changes in MA30- and PR8-infected mice

To identify transcriptional changes in the brain associated with CoV2 and influenza A infections, we conducted bulk RNA-seq analysis of brain tissues from MA30-infected and PR8-infected mice. Analyses were conducted at both the acute (7 DPI for MA30 and 8–9 DPI for PR8 infection respectively) and subchronic (21 DPI for both MA30 and PR8 infection) stages of infection.

As compared to uninfected controls, the brain tissue of MA30-infected mice exhibited upregulation of genes involved in the complement system (e.g., *Cfd* encoding Complement Factor D) and hormone signaling (e.g., *Gh* and *Prl*, encoding growth hormone and prolactin, respectively) during the acute phase of infection (**Supplementary Table 4**). In contrast, genes related to neurotransmitter synthesis and transport, including *Dbh* (dopamine beta-hydroxylase), *Tph2* (tryptophan hydroxylase 2), *Maoa* (monoamine oxidase A), and several serotonin/dopamine transporters (*Slc6a4*, *Slc6a3*, *Slc18a2*), were significantly downregulated (**Supplementary Table 4**; **Supplementary Figure 13A**).

Similarly, brain from PR8 infected mice displayed upregulation of hormone-related genes (*Prl*, and *Gh*) and the prohormone, *Pomc*, encoding Proopiomelanocortin, while genes associated with neurotransmitter production and function (*Dbh*, *Tph2*, *Slc6a3*, *Slc6a4*, *Slc18a2*), collagen remodeling (*Col11a2*), and ECM proteases (*Mmp17*, *Mmp24*) were downregulated, as compared to uninfected mice (**Supplementary Figure 13B**).

Pathway analyses revealed shared upregulation of glucocorticoid receptor regulatory networks and prolactin receptor signaling in both MA30 and PR8 acute phase infections, as well as suppression of cerebellar and medullary markers and dopaminergic neurogenesis pathways (**Supplementary Figures 14**, **15**). Notably, the peroxisome proliferator-activated receptor (PPAR) signaling pathway was uniquely upregulated in MA30-infected mice brain tissue, suggesting virus-specific transcriptional responses (**Supplementary Figure 14A**). Brain tissue of acute PR8-infected mice further shows upregulated growth hormone signaling, and downregulated NaCl dependent neurotransmitter transporter signaling pathway and extracellular matrix organization (**Supplementary Figure 15B**).

Pairwise comparison revealed distinct gene expression profiles in acute MA30-infected and acute PR8-infected mice brain. Specifically, the brain from MA30-infected mice exhibited upregulation of complement (C1ql2), pro-inflammatory (Il18, Ifnar2), and collagen (Col11a2) related genes, whereas hormone genes (*Prl*, *Gh*, *Pomc*) were downregulated (**Supplementary Table 5**; **Supplementary Figure 16**). Pathway analysis further indicated enrichment of the complement cascade, PPAR signaling, IL22 signaling, and collagen degradation in brain of acute MA30-infected mice compared to that of PR8-infected mice (**Supplementary Figure 17A**). No significant downregulated pathways of interest were seen in the pairwise comparison in acute MA30-infected mice brain (**Supplementary Figure 17B**). These acute-phase transcriptomic signatures reflect neuroinflammatory alterations similar to those observed in patients with COVID-19 and may contribute to long-term neurological sequelae of LC (**Figure 5**).

At 21 DPI, brain from MA30-infected mice exhibited persistent transcriptional remodeling compared to uninfected controls (**Supplementary Figures 18**-**20**). Hormonal genes (*Prl*, *Gh*, *Pomc*) remained upregulated, indicating continued dysregulation of neuroendocrine signaling (**Supplementary Table 6**). Concurrently, there was suppression of key neurotransmitter-related genes (*Maoa*, *Tph2*, *Dbh*, *Slc6a4*, *Slc6a3*), suggesting sustained deficits in neurotransmitter biosynthesis, transport, and turnover, that is potentially reflective of neuronal dysfunction or loss (**Supplementary Figure 18A**). In contrast, brains from PR8-infected mice at 21 DPI showed downregulation of *Dbh and Tph2* only, without notable changes in other genes related to neuronal function (**Supplementary Figure 18B**).

Pathway analyses of the brain from subchronic MA30-infected mice showed enrichment in growth hormone receptor, follicle-stimulating hormone, prolactin receptor, and general hormone ligand-receptor interaction pathways (**Supplementary Figure 19A**). In addition, cytokine-cytokine receptor interaction signaling pathway remained upregulated in MA30-infected brain compared to uninfected brain, consistent with persistent low-grade inflammation characteristic of LC (**Supplementary Figure 19A**). Moreover, MA30-infected mice brain has upregulated collagen degradation pathway, in line with our histological findings of microhemorrhage presence at 21DPI, likely reflecting ECM breakdown and vascular basement membrane damage, which may compromise blood–brain barrier (BBB) integrity (**Figure 5**, **Supplementary Figure 19A**). Downregulated pathways included monoamine transport, neurotransmitter transporters, and neurotransmitter clearance signaling pathways (**Supplementary Figure 19B**), in agreement with previous reports linking cytokine storm to altered neurotransmitter dynamics in LC, particularly for serotonin, dopamine, and glutamate (51).

Pathway analysis of PR8-infected brain tissue at 21 DPI revealed upregulated GPCR ligand binding compared to the uninfected brain, suggesting that there was no significant disruption in neurological pathways in the long PR8-infected brain (**Supplementary Figures 20A, B**).

**Figure 6:**
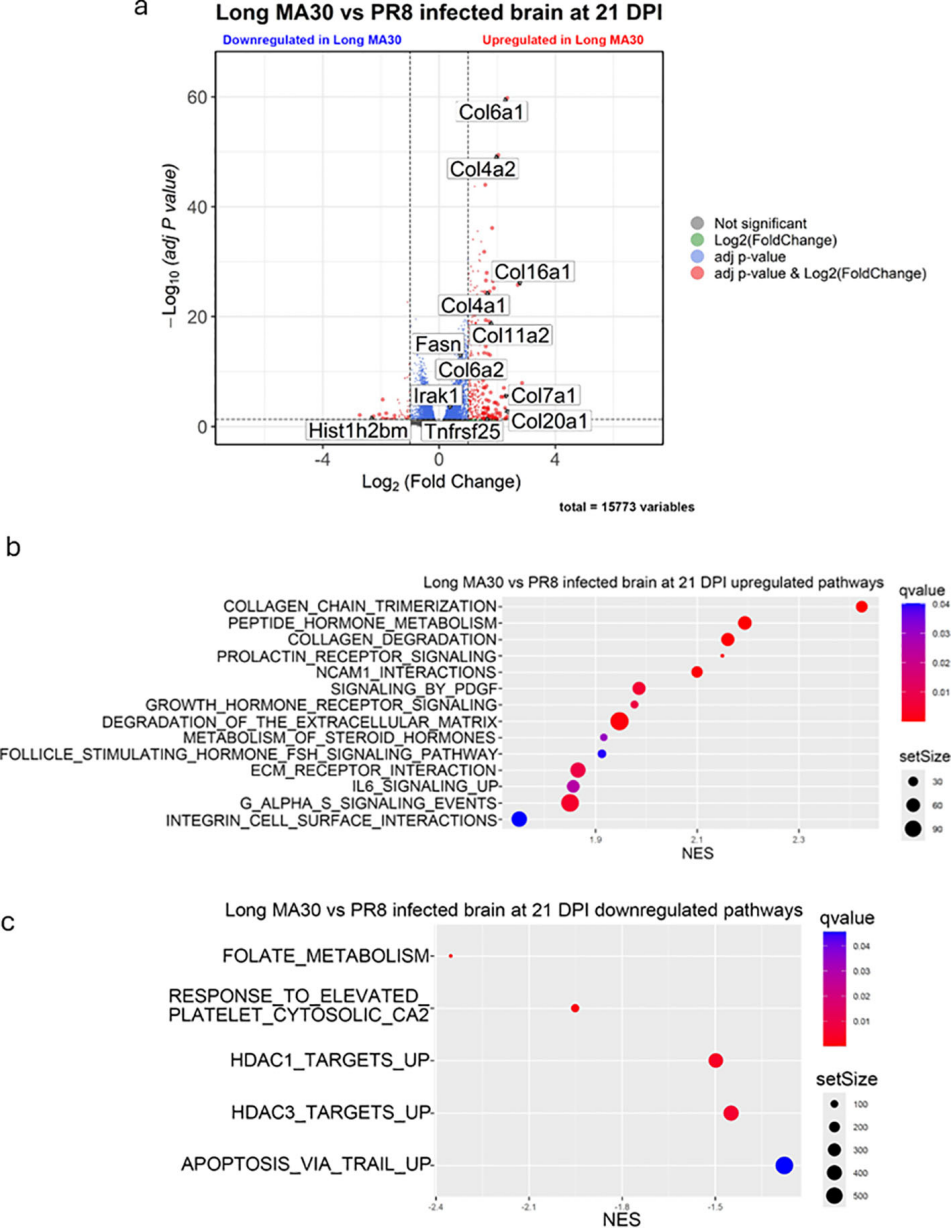
Long-term MA30 infection induces brain-specific transcriptional changes distinct from PR8. **(A)** Volcano plot comparing MA30 (n=6) - and PR8 (n=4) -infected brains at 21 DPI shows MA30-specific upregulation of ECM remodeling genes. **(B)** Enriched pathways in MA30 brains include collagen degradation, hormone signaling and IL6 signaling, indicating sustained neuroinflammation and stress. **(C)** Downregulated pathways involve HDAC, platelet signaling, suggesting impaired brain function. These patterns were not present in PR8-infected brains, highlighting long COVID–specific neurobiological disruption.

Direct comparison of MA30 and PR8 brain transcriptional profiles highlighted virus-specific effects on the brain. Brain from MA30 infected mice maintained elevated expression of hormone-related genes (*Gh*, *Prl*, *Pomc*, *Fshb*) and ECM remodeling genes (*Mmp17*, *Mmp24*, *Col16a1*) (**Supplementary Table 7**; **Figures 5**, **6A**). Furthermore, genes downstream of IL-6 signaling, including *Cebpb* and *Stat3*, were upregulated in subchronic MA30-infected mice brain compared to brain from PR8-infected mice (**Supplementary Table 7**; **Figure 6A**), suggesting sustained inflammatory signaling. Pathway-level comparisons revealed that LC brains maintained enrichment in collagen degradation, ECM receptor interaction, hormone signaling, and IL-6 pathways (**Figure 6B**), whereas histone deacetylase (HDAC)-associated signaling was suppressed (**Figure 6C**). Together, these findings define a durable transcriptional program in subchronic MA30 infection, consistent with LC pathology. This program is characterized by sustained neuroinflammation, impaired neurotransmitter homeostasis, dysregulated hormonal signaling, and persistent vascular remodeling. In contrast, infection with influenza A appears to have a more transient transcriptional response.

## Discussion

Here, we document that young B6 mice (3-month-old) infected with the sublethal doses of mouse adapted CoV2 (or MA30) and influenza (or PR8) strains resulted in persistent lung inflammation and fibrosis by 28 days post-infection. In order to perform head-to-head comparison studies of the maximal respiratory and CNS consequences at histological and transcriptional levels induced by CoV2 and influenza, we have used the sublethal doses of MA30 and PR8 to infect the B6 mice. With those doses, CoV2-infected and influenza-infected mice survived from the acute severe infection with approximately 20% of body weight loss. By this method, we would recapitulate the severe clinical consequences of both viral infections and be able to compare them in the mouse models. Supportively, the respiratory histological changes seen in the both-infected models mirror post-acute lung abnormalities seen in both CoV2 and influenza patients, where fibrotic lesions and reduced lung function may persist for months (19, 21, 22, 52, 53). These findings are consistent with the previously published results showing that influenza A and CoV2 can trigger prolonged immune activation and tissue remodeling in CoV2 and PR8-infected K18-hACE2 model (54) and influenza-infected B6 and Balb/c mice (23, 24, 55). MA10-infected BALB/c and aged B6 mice also showed prolonged lung pathology in BALB/c animals but minimal changes in the B6 mice (31–33). Here, we also observed sex-based differences in weight loss and survival among MA30-infected mice, with females showing improved recovery compared to males. While our study was not designed to dissect these sex-based interactions mechanistically, the clear divergence in outcomes suggests that sex based pathological outcomes may be studied in the MA30 model. Further investigation is warranted to determine how sex influences both acute and long-term pathology during CoV2 infection.

In addition, we also document that KRT5 pod structures overlap with consolidated regions in PR8 infected mice lungs but not in MA30 in both acute (7 DPI) and sub chronic phases (14 and 21 DPI) of the infections. Consistently, our previous studies also demonstrated that histologically, influenza A- but not CoV2-infected K18-hACE2 mice showed extensive proliferation of Krt5+ “pods” that co-stained with stem cell markers Trp63/NGFR in the consolidated lung regions at both 7 and 14 DPI (26). Thus, our findings reported here support and extend our previous findings that CoV2 infection fails to induce nascent Krt5+ cell proliferation in consolidated regions, thereby contributing to incomplete repair of the injured lung (26).

Unlike the lungs, brain pathology was exclusive to MA30 infection. Despite the absence of viral RNA or protein, MA30-infected mice exhibited elevated microhemorrhage frequency at earlier timepoints and glial activation across all timepoints, while PR8-infected mice showed minimal changes. IHC revealed microglial morphological changes and nodular lesions in MA30 brains suggestive of a pro-inflammatory state, alongside astrocyte activation and structural disruption. PR8-infected brains appeared largely indistinguishable from controls but did demonstrate modest gliosis. This difference mirrors clinical findings: neurological complications such as brain fog, fatigue, and cognitive decline are frequently reported after CoV2 but rarely after influenza infection. MRI and PET imaging studies in LC patients have demonstrated glial activation and altered brain perfusion without detectable virus, pointing toward systemic immune or vascular mechanisms rather than direct neuroinvasion (16, 56). Similar to published data using other pathogens, the MA30 model recapitulates this non-viral, inflammation-driven neuropathology and thus provides a valuable platform for mechanistic exploration (57, 58). Considering that B6 mice is a widely used strain for investigating the pathogenesis of human diseases (26, 59) due to the availability of gene-specific knockouts on this B6 background (60), our results reported here underscore that MA30-infected B6 mice is applicable for studying the pathogenesis of LC especially for COVID19-induced respiratory and central nervous system sequelae.

Bulk RNA sequencing analysis of the lungs and brains collected from MA30- and PR8-infected mice at either acute or sub chronic phases reveals the transcriptomic trajectories, which explain the histological abnormalities in the both viral infected lungs and CoV2-infected brains in these mice. In PR8-infected lungs, transcriptomic signatures indicated active recovery, with upregulation of keratin family genes (*Krt5*, *Krt6a*, *Krt15*, *Krt17*) and interferon-stimulated genes (*Stat1*, *Ifng*, *Irf7*), consistent with a regenerative response. This was supported by the presence of KRT5+ pod structures in PR8 lungs and aligns with previous reports of basal progenitor–driven epithelial repair after influenza infection (50, 61). In contrast, MA30-infected lungs lacked keratin upregulation and instead showed persistent activation of complement, coagulation, and fibrotic pathways, including upregulation of *Fga*, *FggSerpine1b*, *C1qa*, *C3ar1*, and pro-inflammatory cytokines such as *Cxcl10*, *Il1b*, and *Tnf*. Pathway analyses revealed strong enrichment for ECM remodeling, and coagulation cascades, alongside downregulation of epithelial junction and keratinization-related genes. These divergent profiles suggest that while both viruses cause histological lung injury, PR8 promotes regenerative repair, whereas MA30 drives sustained immune activation and impaired epithelial healing. Critically, our data points to a prominent role for the IL-6 signaling axis in LC-associated neuroinflammation. Genes such as *Cebpb* and *Stat3*, well-known transcriptional targets of IL-6, remained upregulated in MA30-infected mice brains at 21 DPI compared to PR8. At the pathway level, IL-6 signaling enrichment was unique to MA30-infected mice brains, supporting the hypothesis that sustained low-grade cytokine signaling persists beyond viral clearance. Given the central role of IL-6 in gliosis, synaptic remodeling, and sickness behavior, this may represent a key driver of post-viral neurological dysfunction. Consistently, by using autopsy platform allowing direct spatial correlation of focal neuropathological and microglial phenotype changes with central and systemic inflammation, a recent study documents that microglia dysfunction, neurovascular inflammation and focal neuropathologies are linked to IL-6-related systemic inflammation in COVID-19 (62).

Sustained upregulation of collagen degradation signaling in MA30-infected mice brains is seen in the acute and subchronic phases, including genes such as *Mmp17*, *Mmp24*, and *Col16a1* remained elevated even at 21 days post-infection. This transcriptional signature aligns with histological evidence of microhemorrhages, suggesting that CoV2 infection leads to chronic extracellular matrix (ECM) breakdown and basement membrane compromise, potentially contributing to long-term blood–brain barrier (BBB) dysfunction and capillary fragility. In contrast, PR8-infected mice exhibited only transient ECM changes, without persistent activation of collagenase pathways or overt microvascular pathology. In addition, enzymes essential for neurotransmitter biosynthesis and clearance—including *Maoa*, *Tph2*, *Dbh*, *Slc6a3*, and *Slc6a4*—were consistently repressed during both the acute and subchronic phases. These changes suggest impaired neurotransmitter turnover and synaptic function, which may underline the cognitive dysfunction, fatigue, and neuropsychiatric symptoms frequently reported in LC patients. While PR8-infected mice also transiently suppressed neurotransmitter-associated genes, these changes were less persistent and not accompanied by significant disruption in associated pathways. Together, these findings delineate a durable and virus-specific transcriptional program in the CNS following CoV2 infection in mice, distinct from influenza. LC is marked by persistent neuroinflammation, hormonal imbalance, neurotransmitter dysregulation, and vascular remodeling, all of which may synergize to impair brain homeostasis and function. These signatures mirror key aspects of the human post-acute sequelae of CoV2 (41, 63), and provide a mechanistic foundation for therapeutic targeting of IL-6, ECM remodeling, and monoaminergic circuits in LC–associated neurological disease.

Our comparative transcriptomic analysis of MA30- and PR8-infected mice brains demonstrate that MA30 infection drives a more persistent and multifaceted transcriptional dysregulation in the brain hormonal pathways. Both PR8- and MA30-infected mice acutely induced hormone-related genes (*Gh*, *Prl*, *Pomc*), consistent with systemic inflammatory stress responses. However, by 21 DPI, MA30-infected mice brains retained elevated expression of these hormones and further showed upregulation of Follicle-Stimulating Hormone (FSH) signaling, growth hormone receptor, and prolactin receptor pathways. This finding is consistent with the growing understanding of the dysfunction of the pituitary and endocrine system in the pathogenesis of Long COVID-19 syndrome (64–66). These findings suggest a virus-specific disruption of the hypothalamic–pituitary axis, with MA30 infection eliciting chronic neuroendocrine dysregulation that may modulate immune–neural crosstalk and glial activation over time.

Together, these findings emphasize that persistent lung pathology is a shared feature of both CoV2 and influenza infection, while brain pathology is a unique and durable consequence of CoV2. This highlights the value of comparative post-viral models in distinguishing shared from virus-specific disease features. Future work should explore the molecular signals that drive CNS involvement in the absence of direct infection, as well as interventions that enhance epithelial repair or reduce chronic inflammation across organ systems. The MA30 and PR8 model provides a tractable platform to investigate these mechanisms and test novel therapies for LC and long term influenza post-viral syndromes.

## Data Availability

Bulk RNA and Single-cell RNA sequencing data reported in this paper have been deposited to the GEO database and is available publicly under the accession number: GSE315893.
