# Supplementary material for: Characterization of subchronic lung and brain consequences caused by mouse-adapted SARS-CoV-2 and influenza A infection of C57BL6 mice
Source: Front Immunol. 2026 Feb 25;17:1755141. doi: 10.3389/fimmu.2026.1755141 (PMC12975924; doi:10.3389/fimmu.2026.1755141)
Supplement: Supplementary file 1 [file DataSheet1.pdf]

Supplement

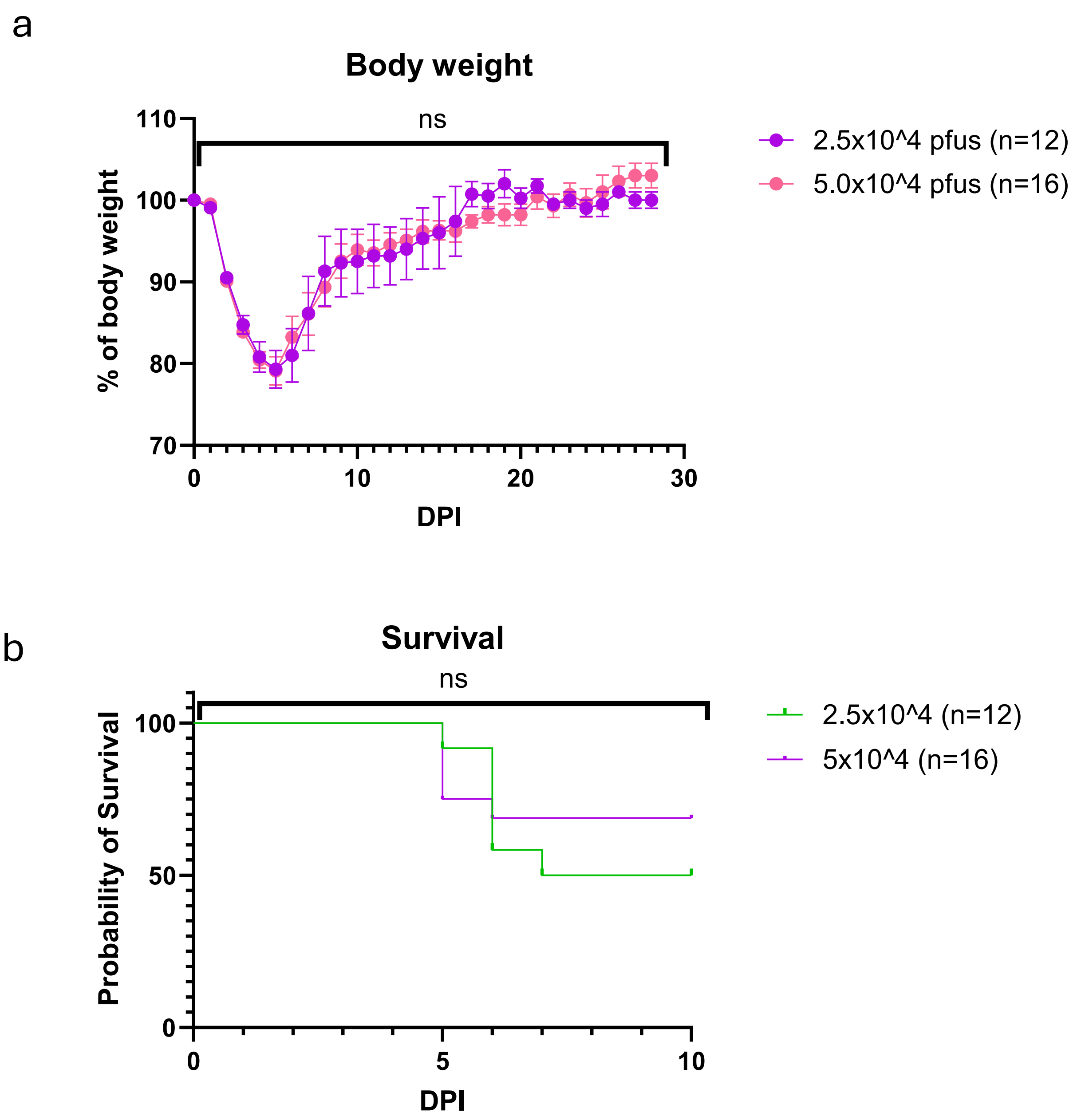

Supplementary Figure 1: Inoculation with different doses of SARS-CoV-2 MA30 (MA30) do not induce pathological changes a) Average percentage body weight was compared after 28 days post infection (DPI) with MA30 and there was no significant body weight differences observed between inoculation doses b) Survival was compared for 10 days post infection (DPI) with MA30 and there were no significant survival differences between inoculation doses.

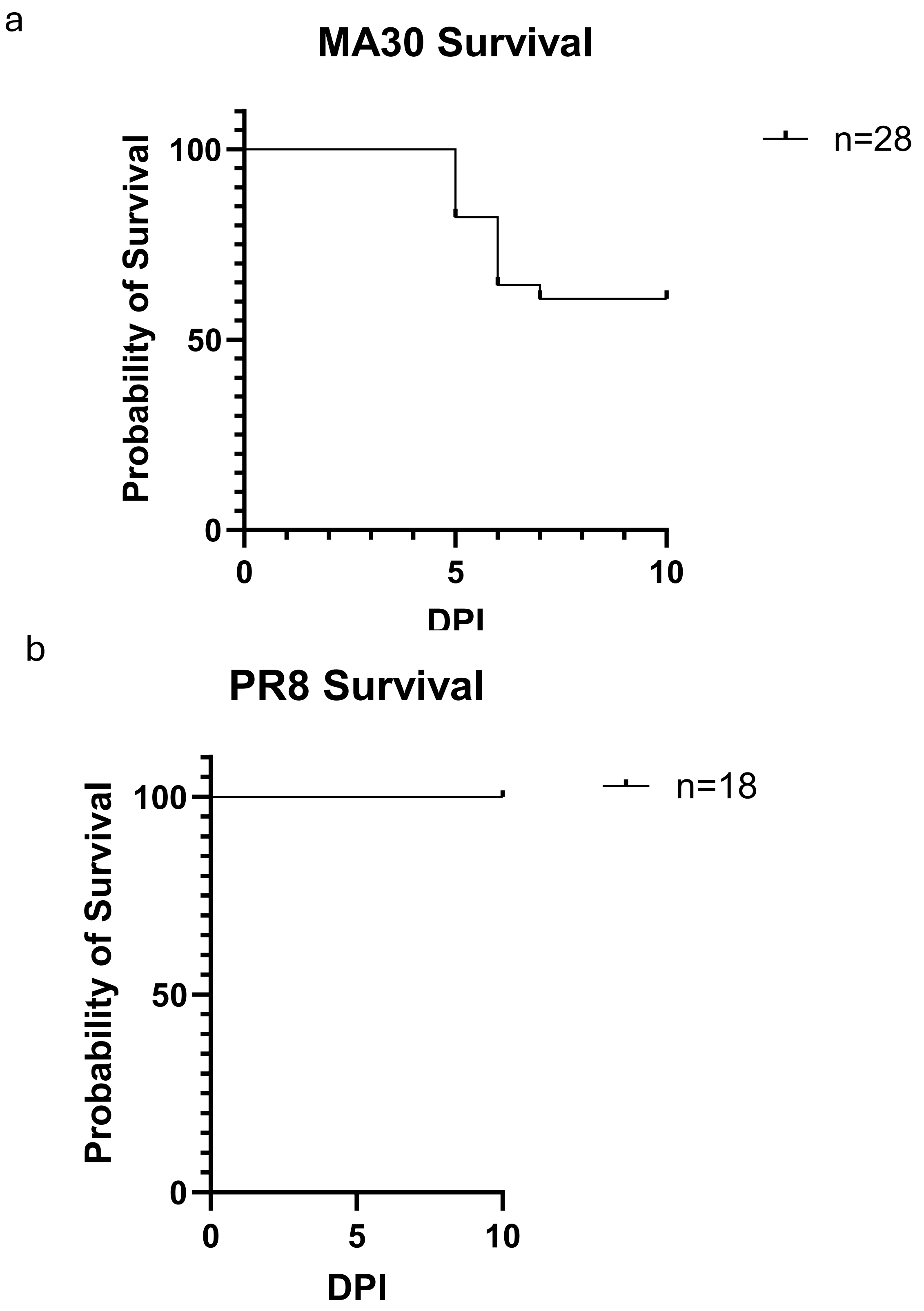

Supplementary Figure 2: Survival curves for C57BL/6 mice infected with SARS-CoV-2 MA30 or influenza PR8. No deaths were observed in PR8-infected animals. MA30 infection resulted in approximately 40% mortality by 7 DPI.

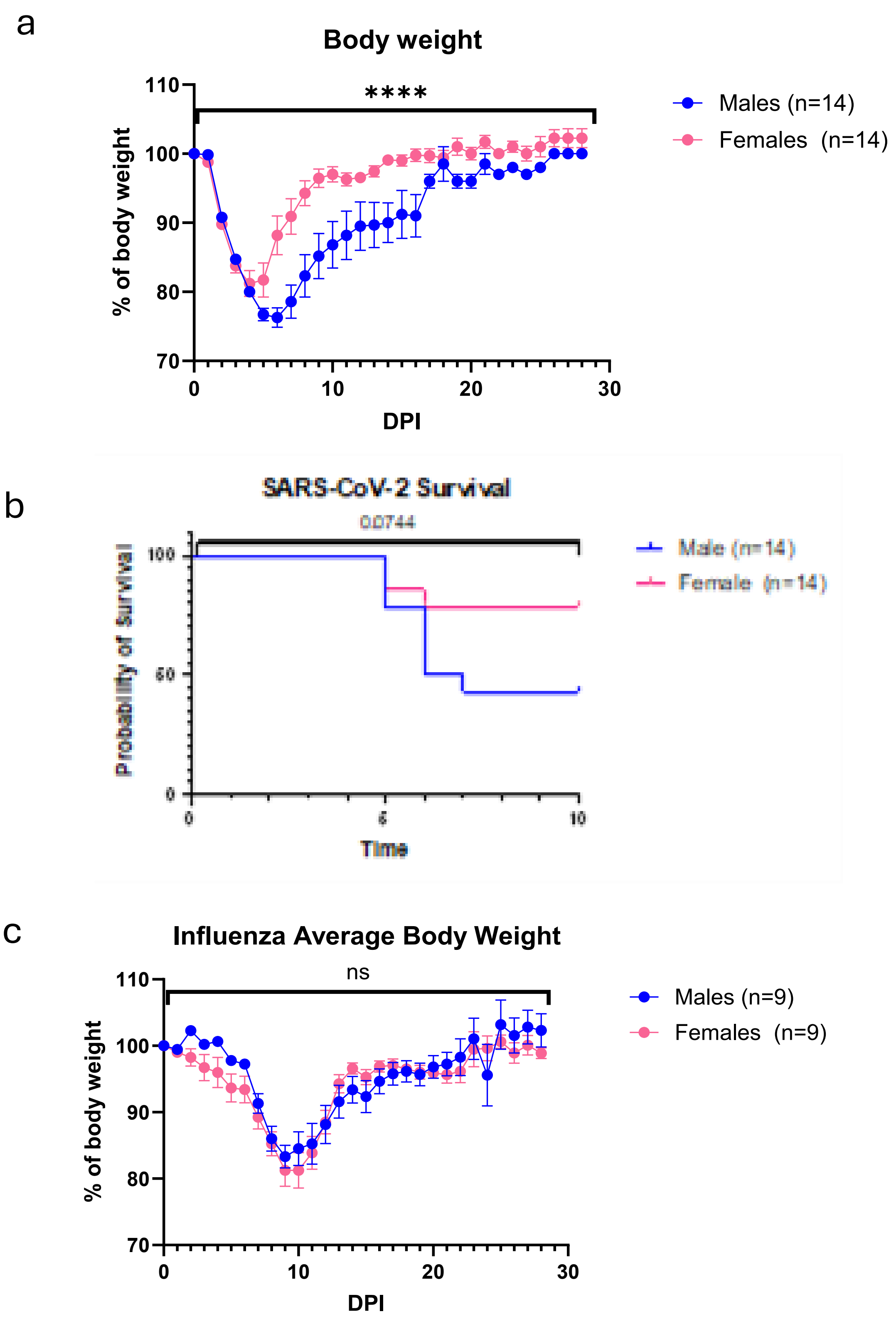

- **Supplementary Figure 3. Sex differences in disease severity following MA30 or PR8 infection.** **(a, b)** Percentages of body weight after MA30 infection (Combined doses $2.5x10^4$ and $5.0x10^4$ TCID50). Male mice infected with MA30 showed greater weight loss and higher mortality compared to females. **(c)** No sex-based differences were observed after influenza PR8 infection (50 PFU).

a

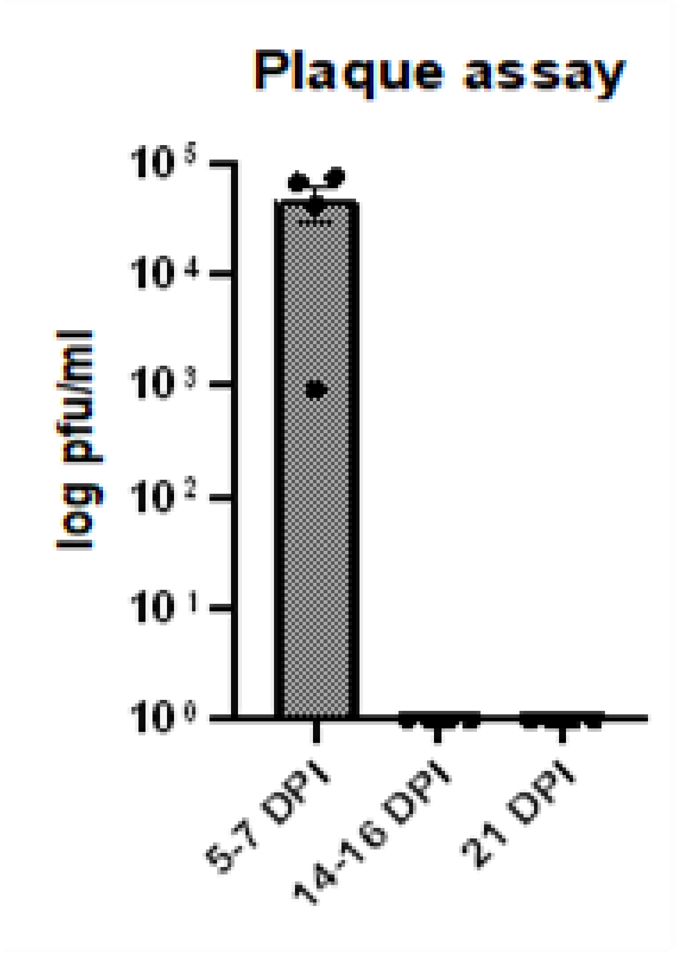

Supplementary Figure 4: Plaque assay results from lung tissue of MA30-infected mice at various timepoints. Replicating virus was not detected 14 or 21 DPI.

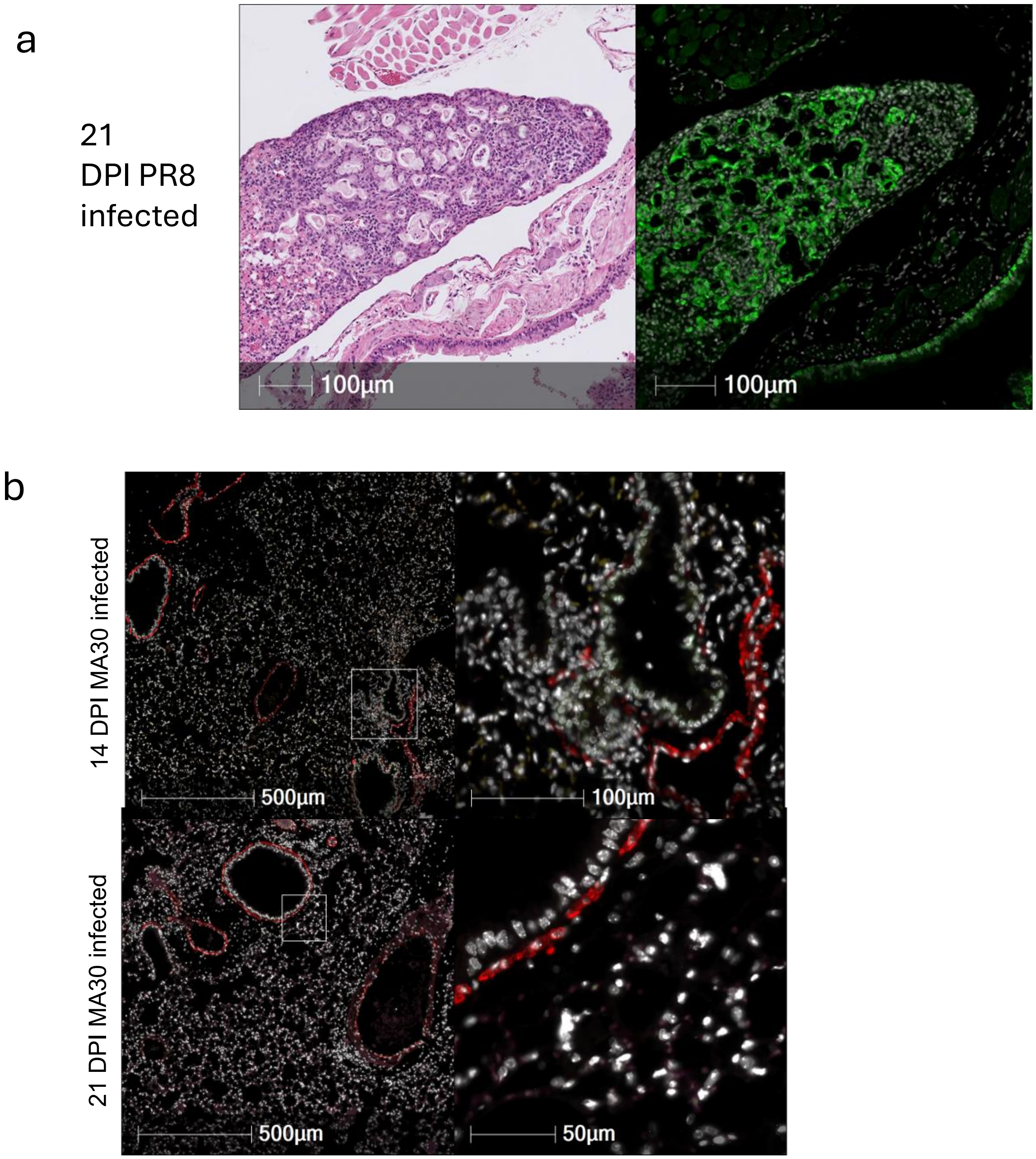

- **Supplementary Figure 5:** KRT5 staining in MA30- and PR8-infected lungs at multiple timepoints shows KRT5+ pod formation only in PR8 lungs at 21 DPI but not in MA30 at 14 or 21 DPI.

a

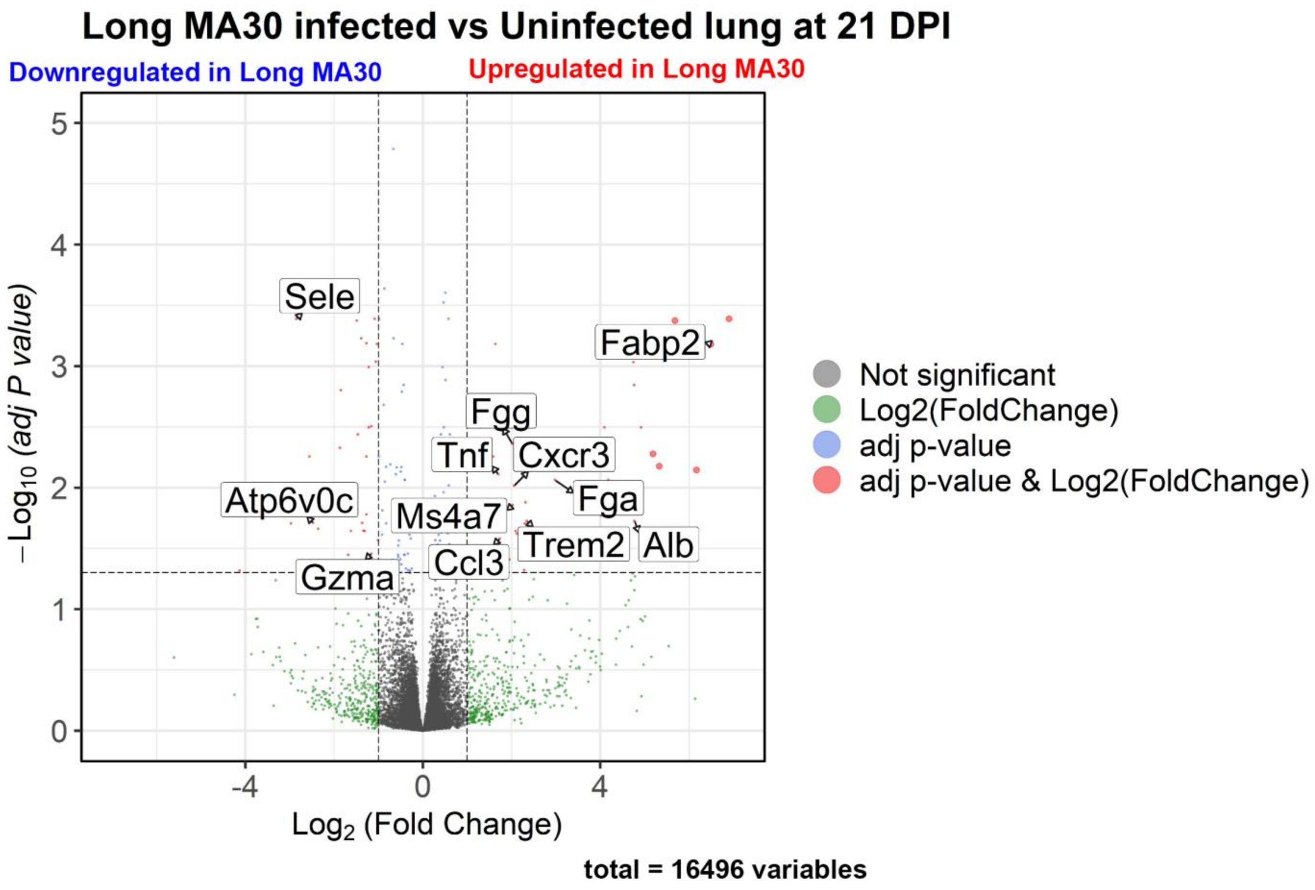

b

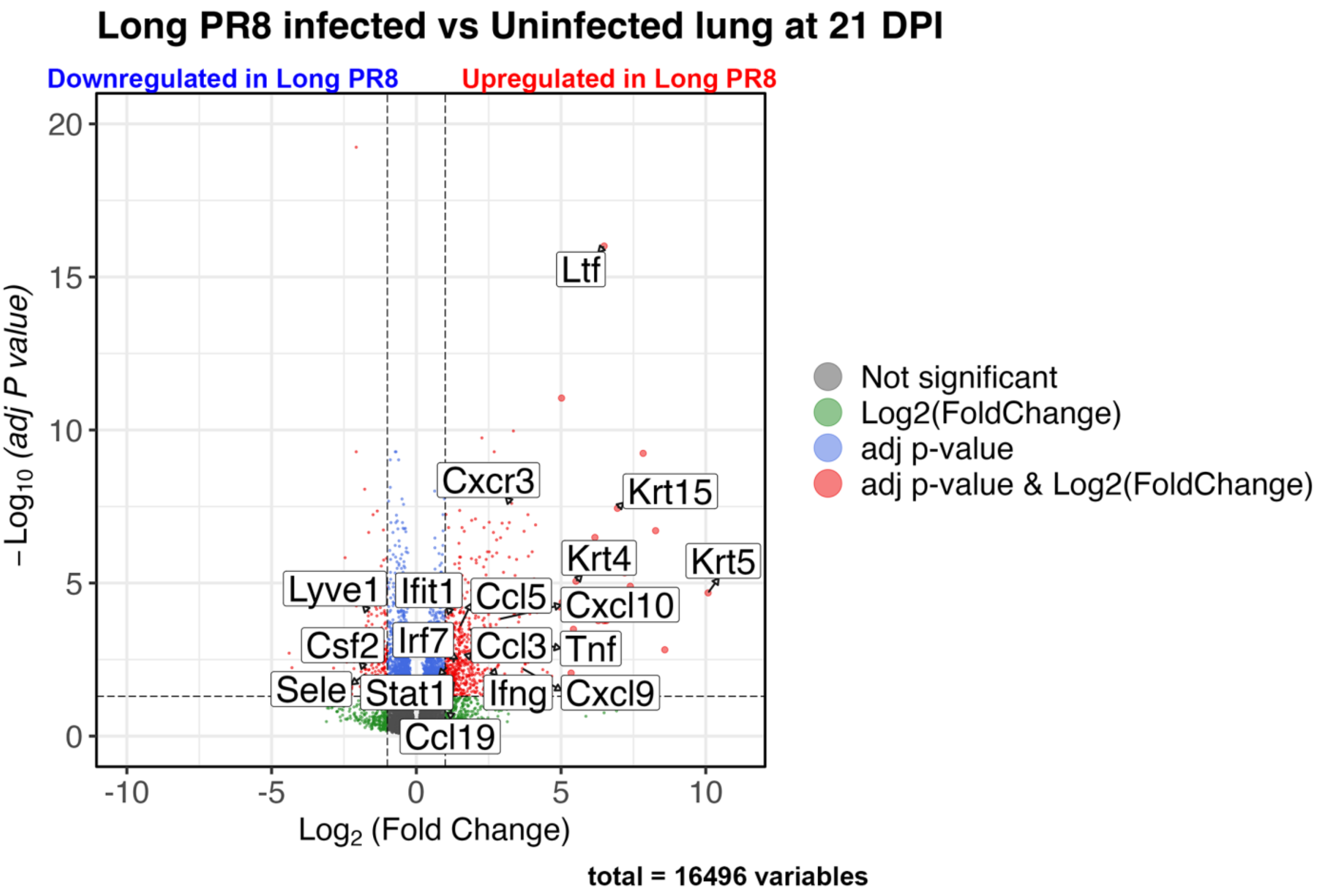

Supplementary Figure 6: Volcano plot showing selected differentially expressed genes in **a)** MA30-infected lung and **b)** PR8-infected lung at 21 DPI compared to uninfected lung.

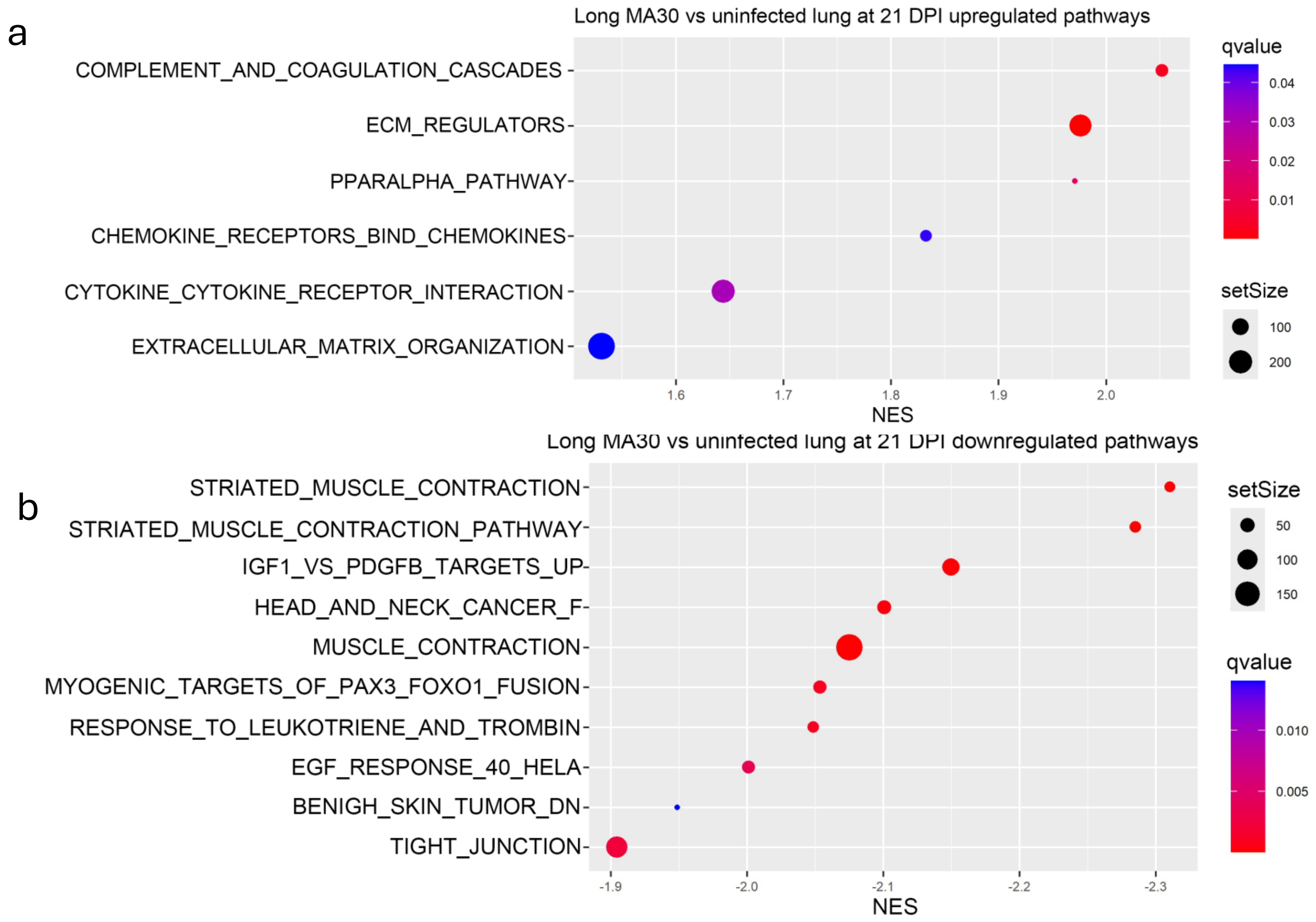

Supplementary Figure 7: Pathway enrichment in MA30-infected lungs at 21 DPI reveals **a)** upregulation of complement activation, coagulation, and fibrotic signaling and **b)** downregulation of TIGHT junctions.

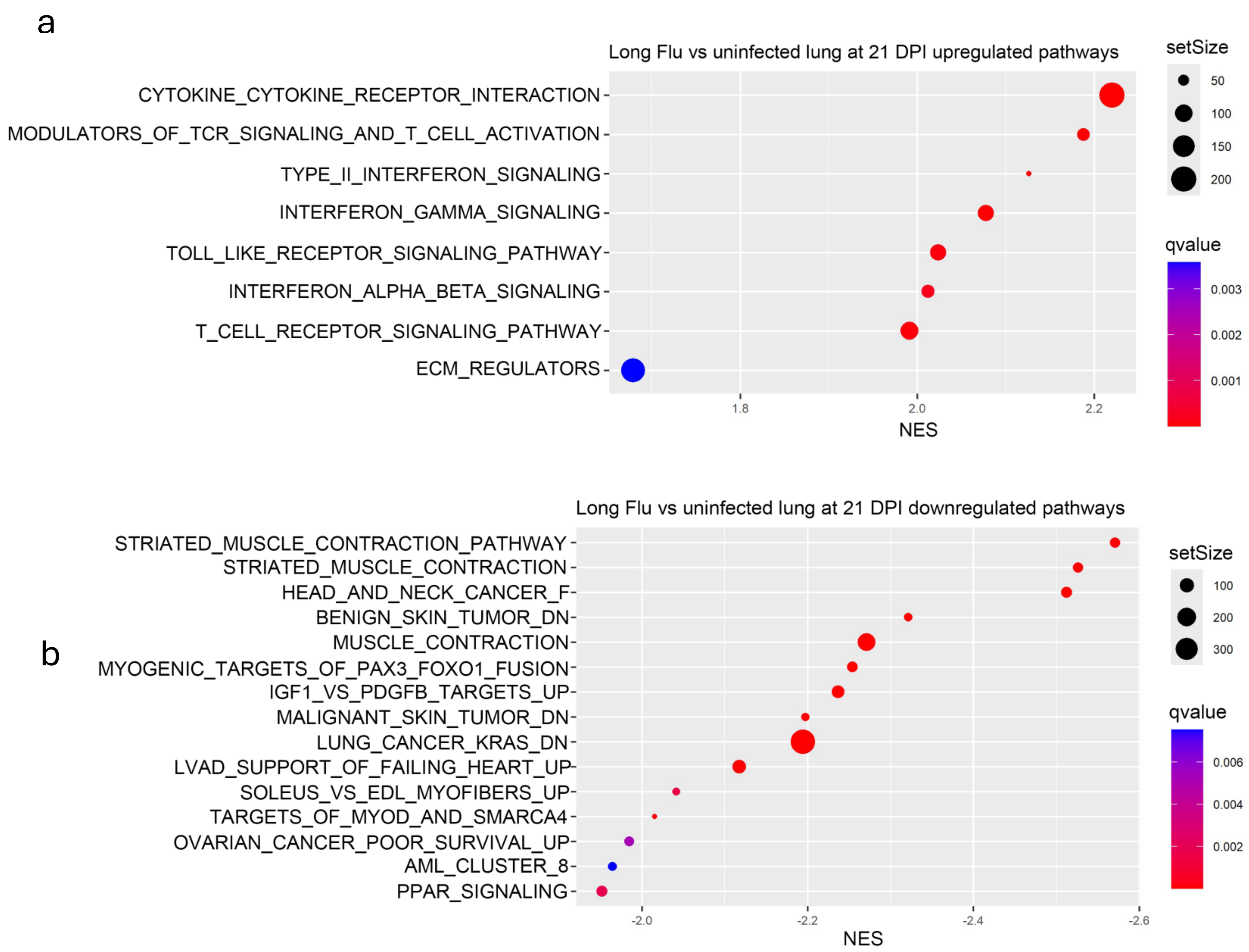

Supplementary Figure 8: Pathway enrichment in PR8-infected lungs at 21 DPI shows continued **a)** upregulation of toll like receptor, cytokine-cytokine receptor interaction, T cell activation, IFN-alpha signaling, IFN-beta signaling, IFN-gamma signaling pathways, and ECM receptor interaction and **b)** down-regulation of muscle contraction, and PPAR signaling.

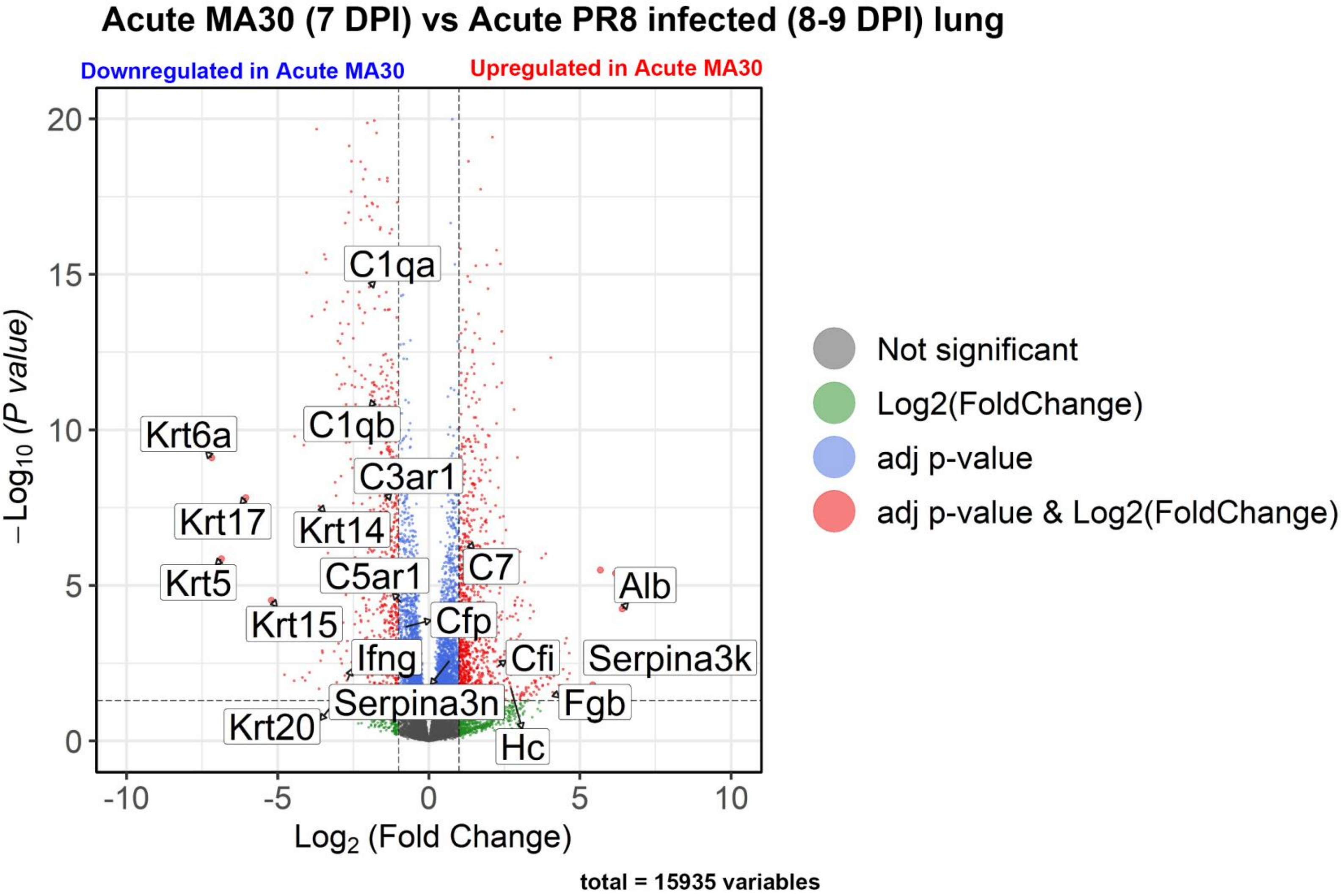

Supplementary Figure 9: Volcano plots from acute MA30-infected (7 DPI) and acute PR8-infected (8-9 DPI) lung RNA-seq reveal upregulated complement activation and coagulation pathway, and downregulated keratin related genes and IFN-gamma responses in acute MA30-infected lung versus acute PR8-infected lung .

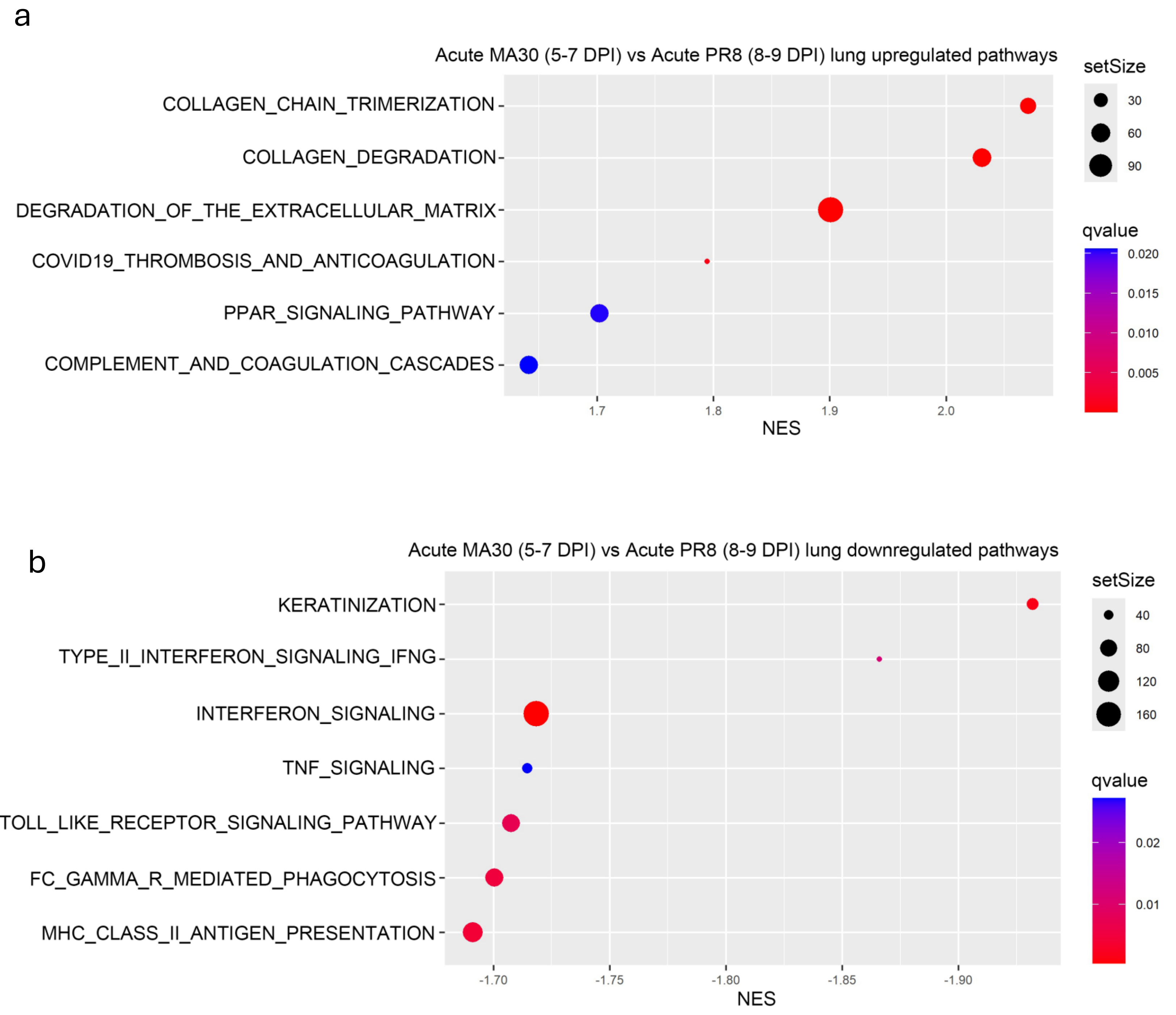

Supplementary Figure 10: Direct comparison of acute MA30-infected (7 DPI) versus acute PR8-infected (8-9 DPI) lungs highlights **a)** upregulated complement and coagulation cascade, PPAR signaling, thrombosis and anticoagulation, and collagen degradation **b)** downregulated MHC class II antigen presentation, toll like receptor signaling, TNF signaling, interferon gamma signaling, and keratinization signaling pathways in acute MA30-infected lungs.

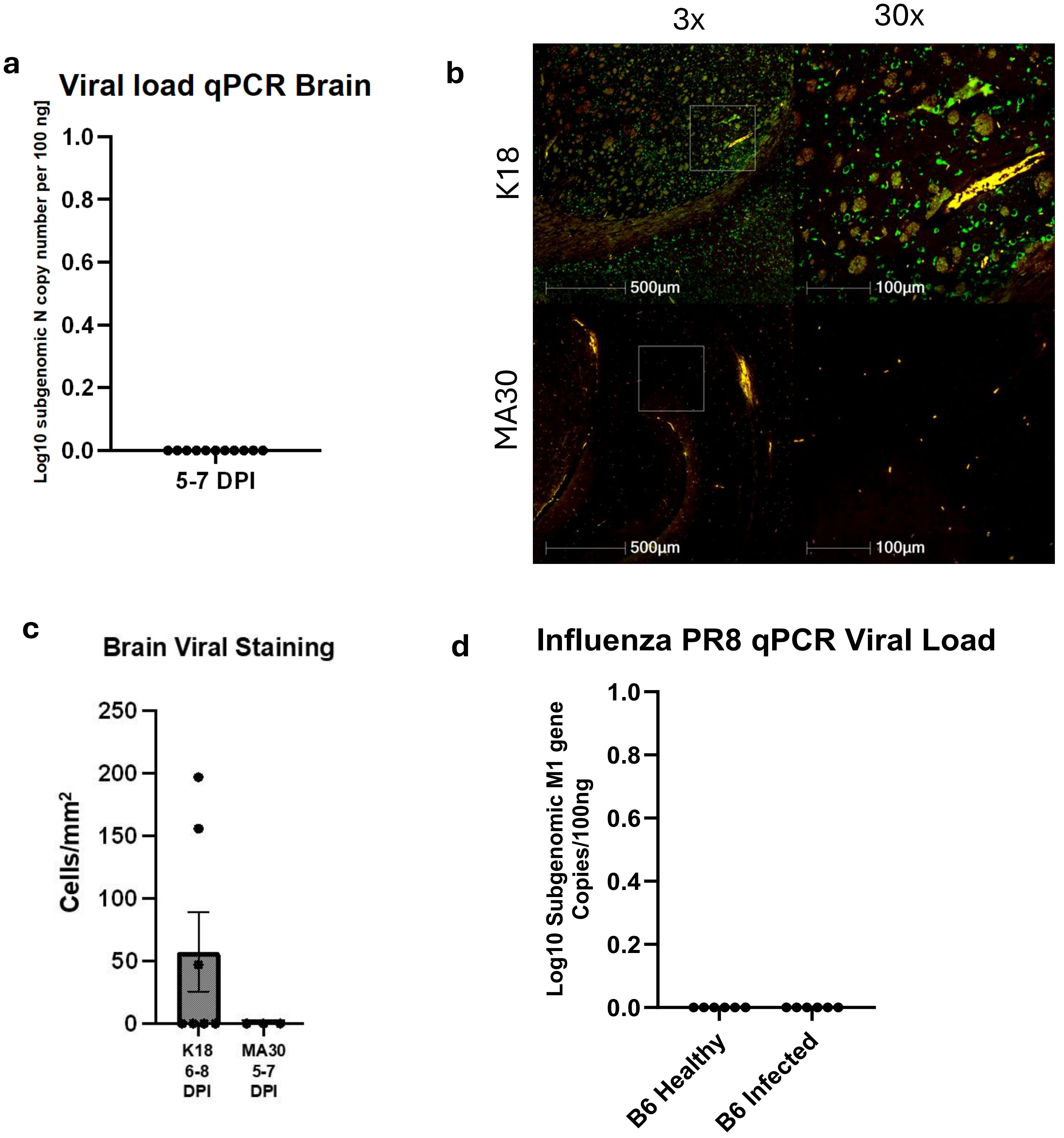

Supplementary Figure 11. Viral detection. Viral detection in MA30-infected mice. RT-qPCR for detecting subgenomic RNA copies of MA30 in the brains of infected mice **(a)** and IHC **(b, c)** show viral antigen in K18 but not MA30-infected mice, confirming lack of brain infection in the MA30 model. **(d)** Viral subgenomic detection by qPCR for monitoring M1 gene RNA copies in the brains of B6 healthy controls (no PR8-infected) and PR8-infected mice at 7 – 9 DPI, confirming lack of brain infection during Influenza A infection.

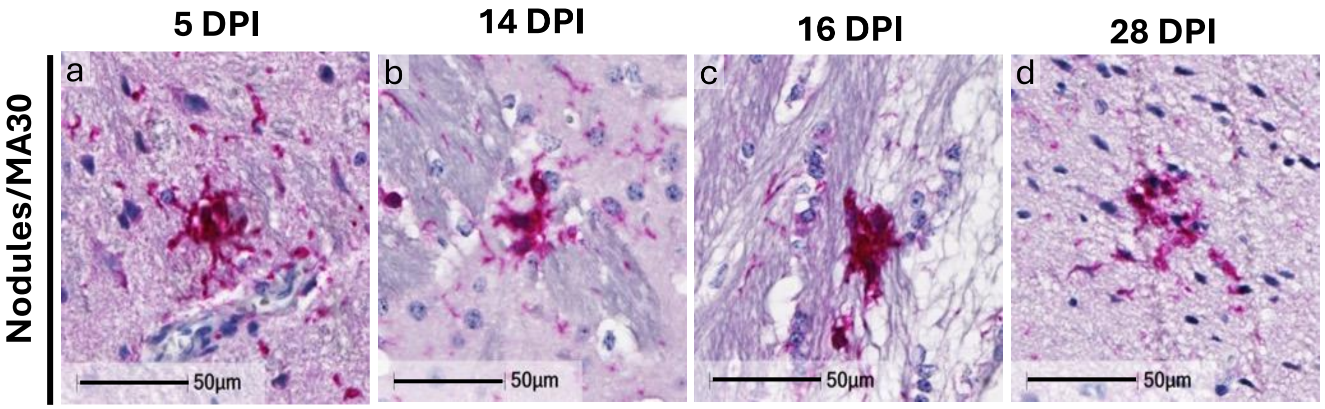

e

Nodular Lesions					
Infection Status	non-infected	5-7, 9 DPI	14-16 DPI	21 DPI	28 DPI
non-infected	0				
MA30-infected		2	3	0	1
PR8-infected		0	0	0	0

Supplementary Figure 12 : Nodular lesions in a subset of MA30-infected mice. Representative images of MA30-infected mice exposed rare nodular lesions, indicative of significant inflammation (a-d). This was observed in six of the twenty-eight MA30-infected animals, across all time points (e). Lesions were not localized to any specific brain region, nor found in brains of non-infected controls or PR8-infected mice.

a

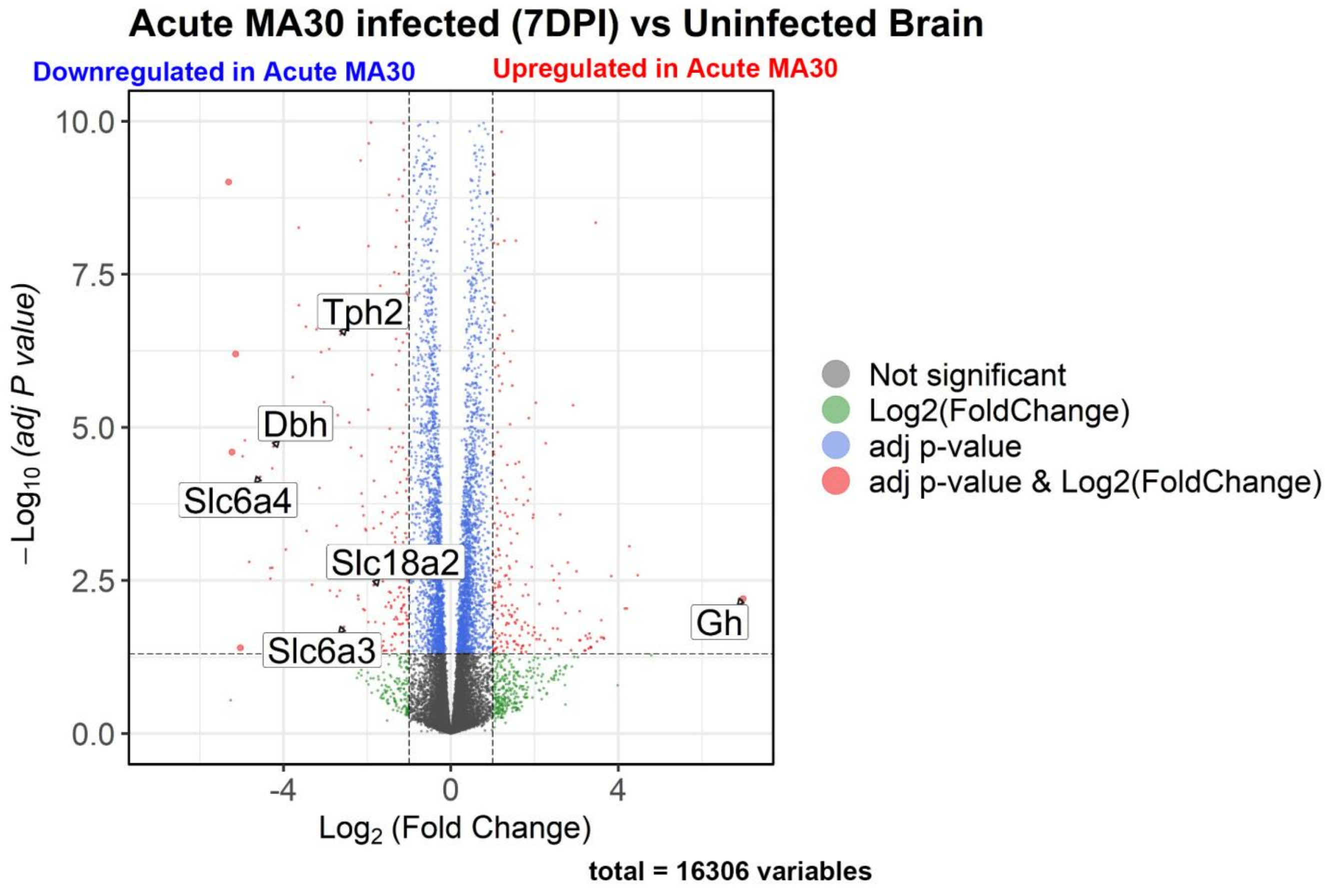

b

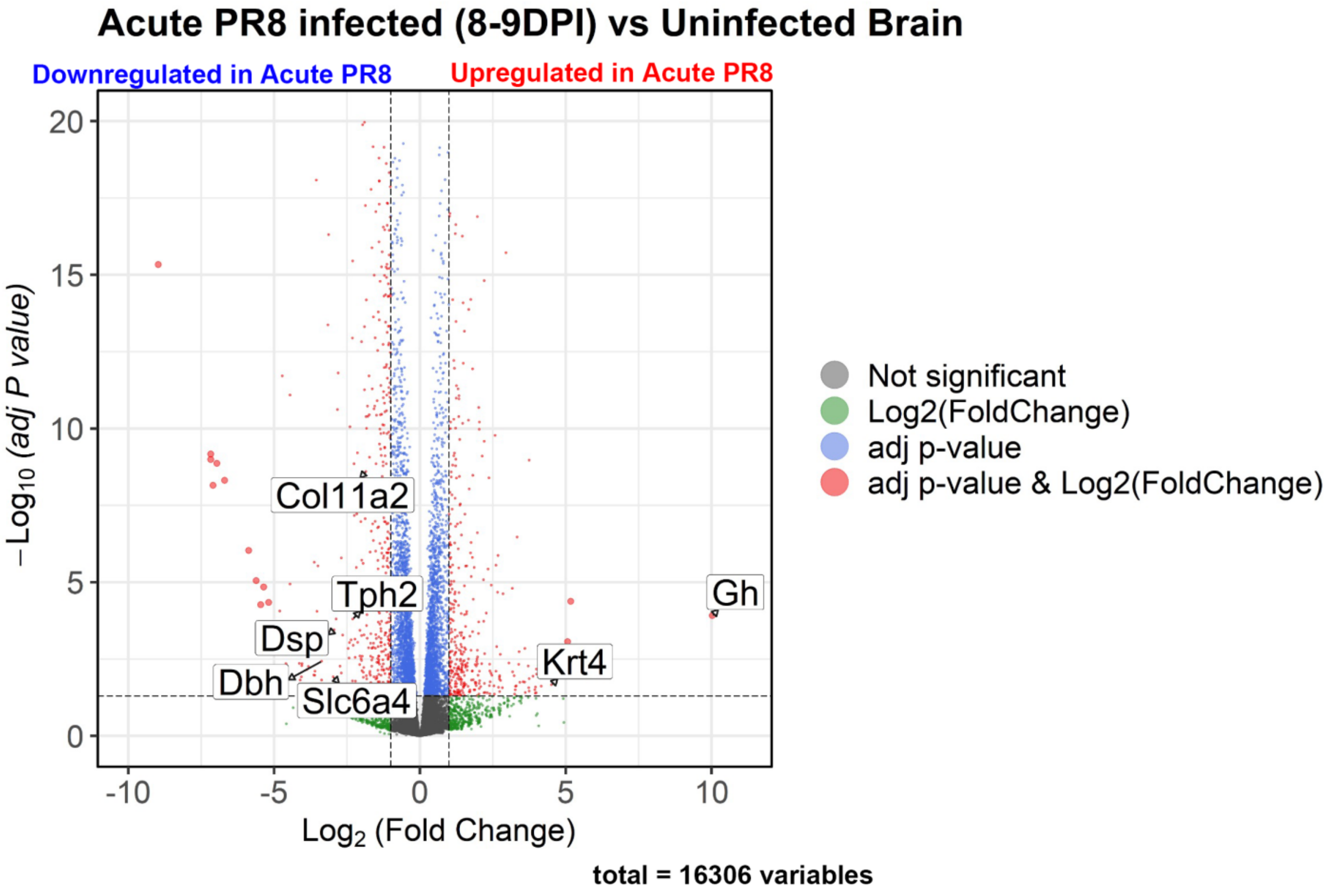

Supplementary Figure 13: Volcano plot comparing acute MA30-infected (7 DPI) and uninfected brain RNA-seq at 7 DPI shows distinct transcriptional responses at early infection.

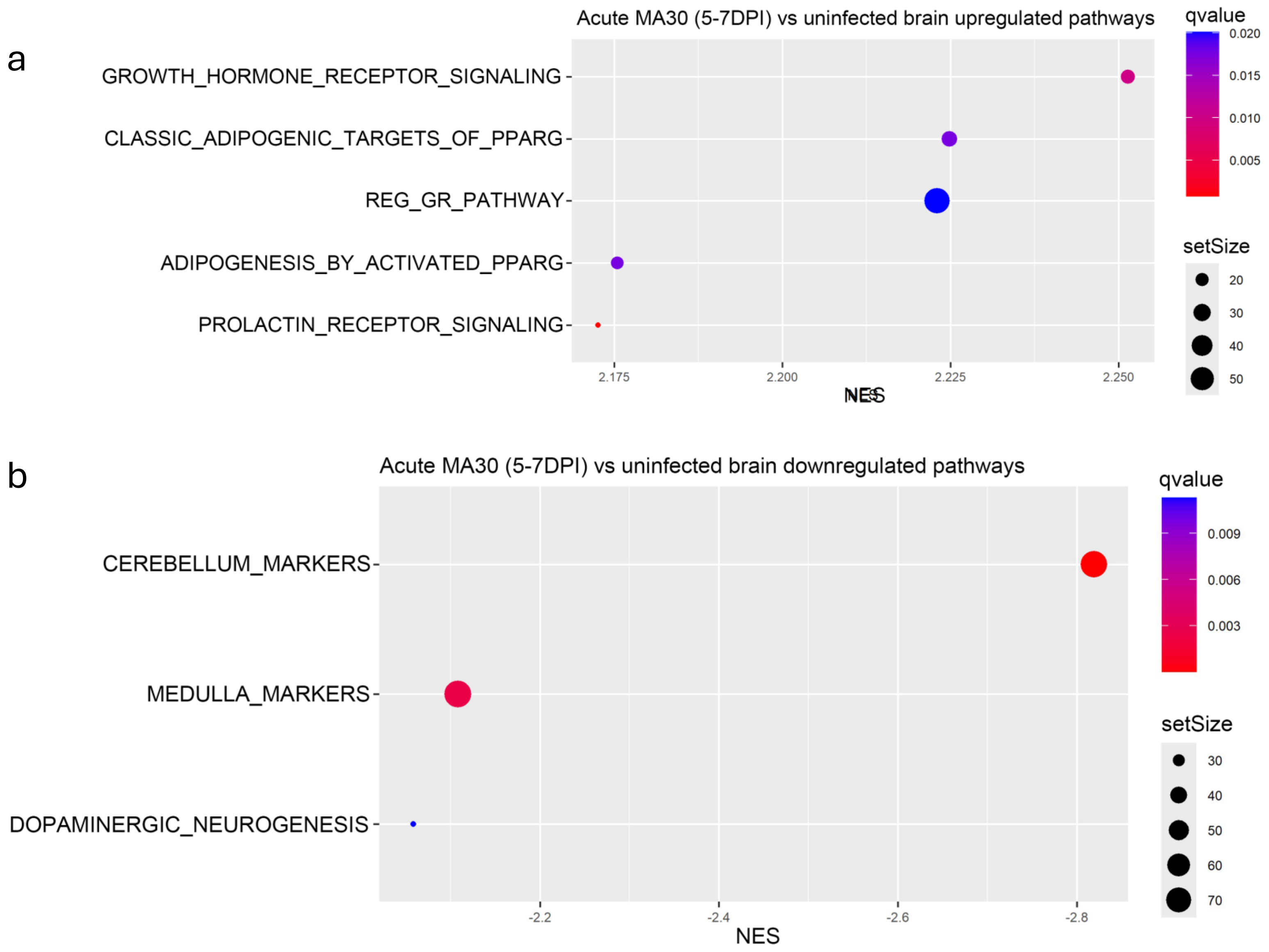

Supplementary Figure 14: Pathway analysis of acute MA30-infected brain at 7 DPI shows **a)** upregulation of glucocorticoid signaling, prolactin signaling, and PPAR-gamma signaling pathways and **b)** downregulation of dopaminergic neurogenesis signaling pathways compared to uninfected brain.

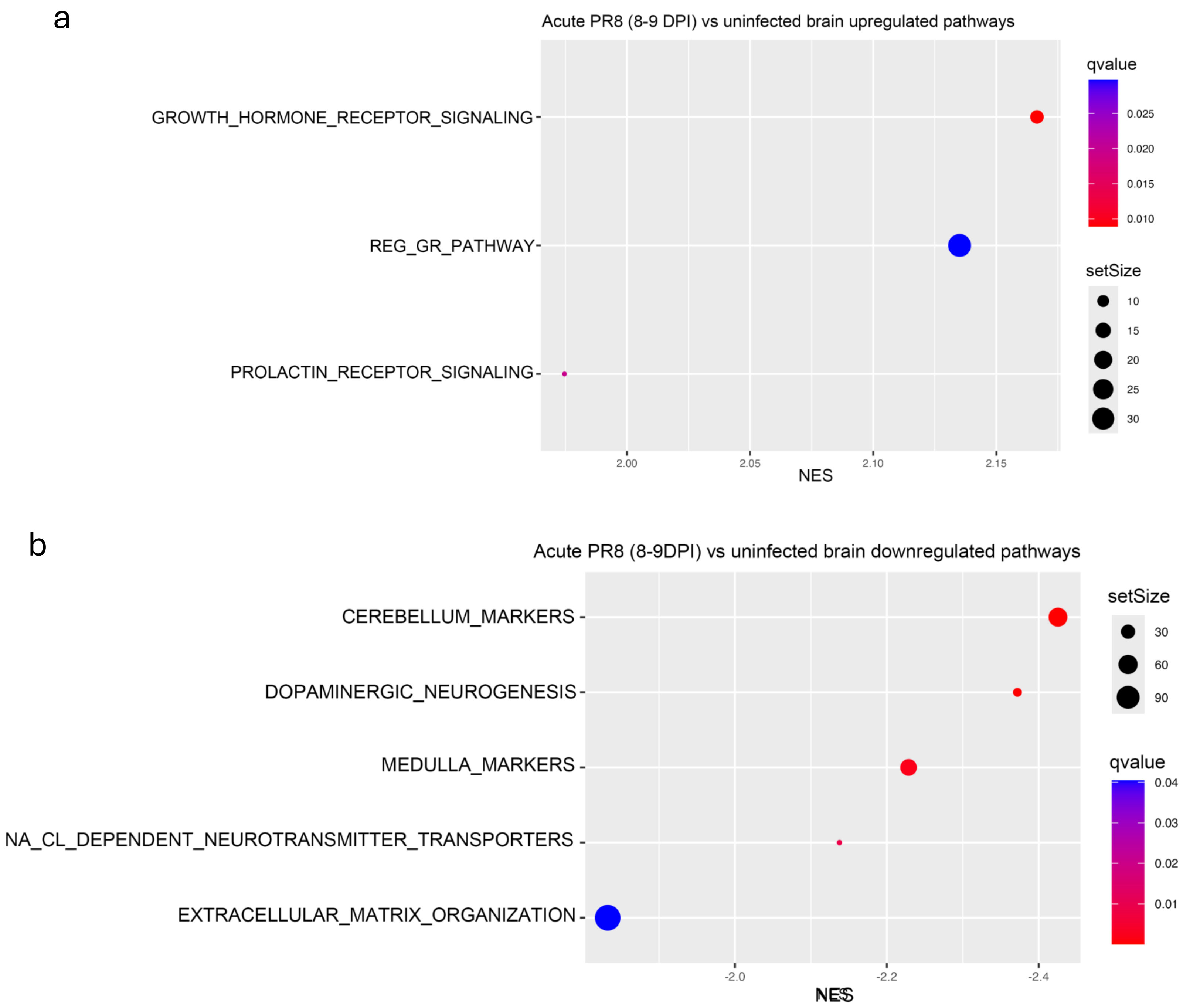

Supplementary Figure 15: Pathway analysis of acute PR8-infected brain at 8-9DPI show **a)** upregulated growth hormone receptor signaling, prolactin receptor signaling, and glucocorticoid receptor signaling and **b)** downregulated dopaminergic neurogenesis, NaCl dependent neurotransmitter transporters and extracellular matrix organization signaling pathway compared to uninfected brain.

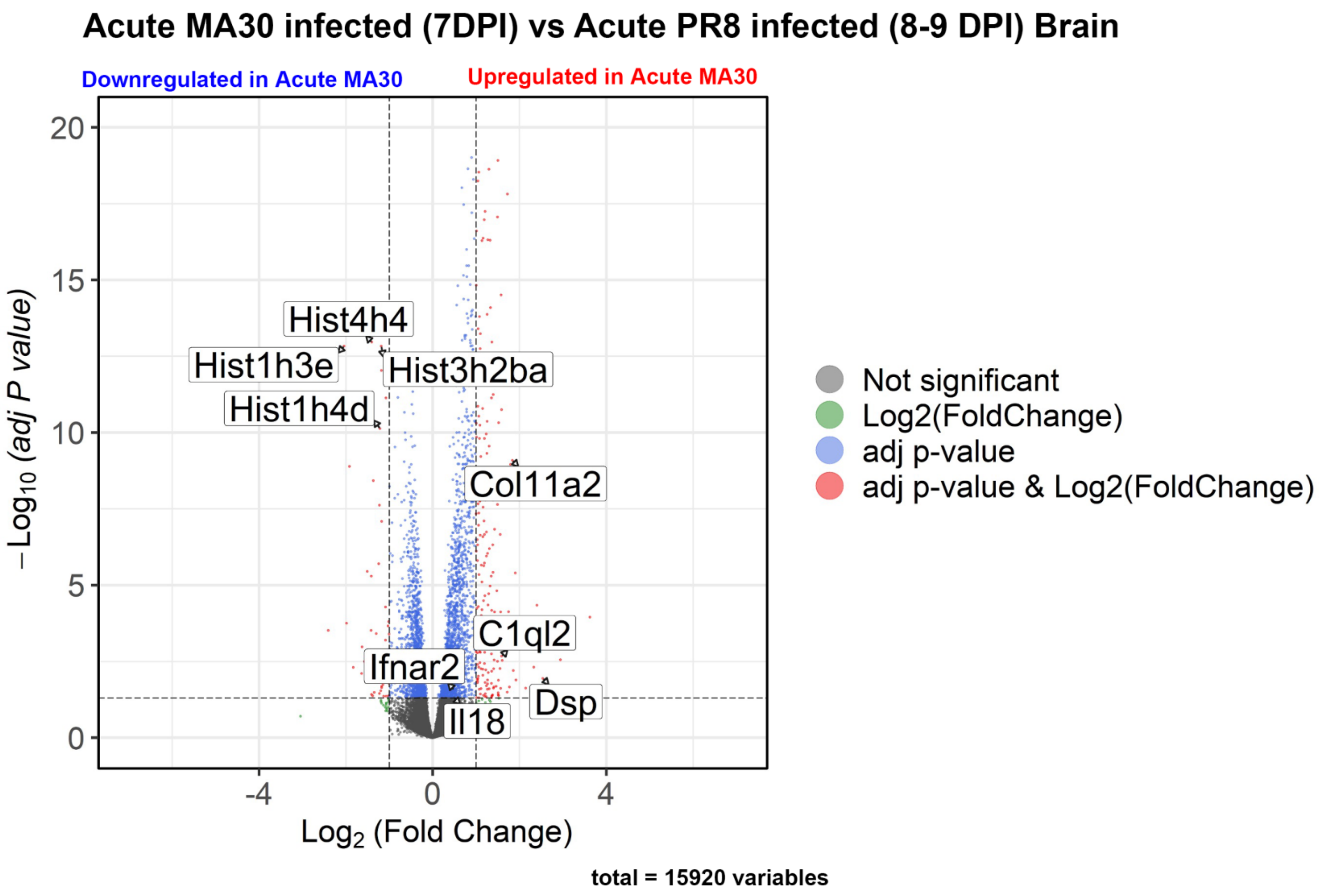

- **Supplementary Figure 16:** Volcano plot comparing acute MA30-infected brain (7 DPI) and acute-PR8 brain (8-9 DPI) shows distinct transcriptional responses early after infection.

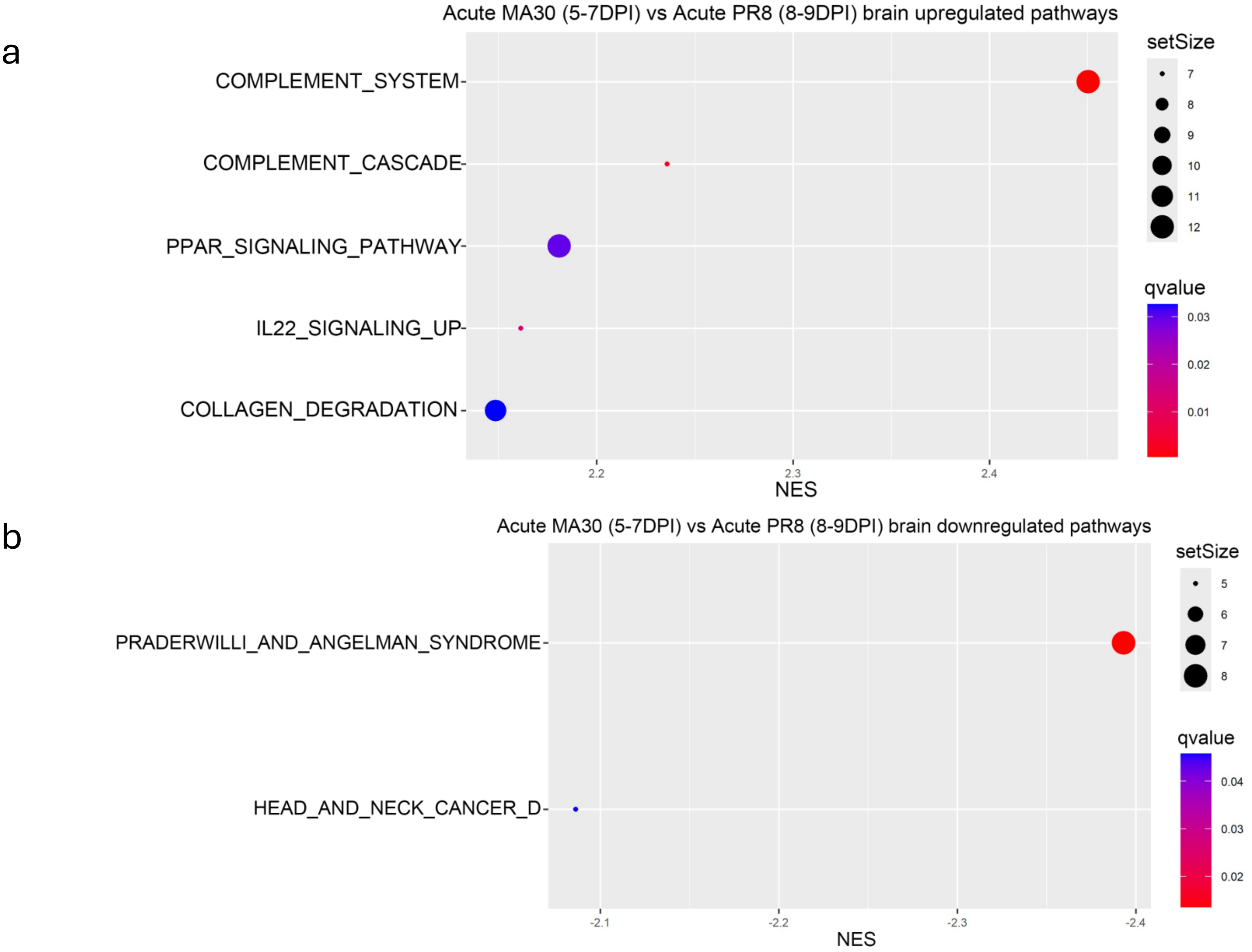

Supplementary Figure 17: Pathway analysis of acute MA30-infected (7 DPI) vs acute PR8-infected brains (8-9 DPI) shows upregulated enrichment of complement activation, IL22 signaling, and collagen degradation pathways in Acute MA30-infected brain.

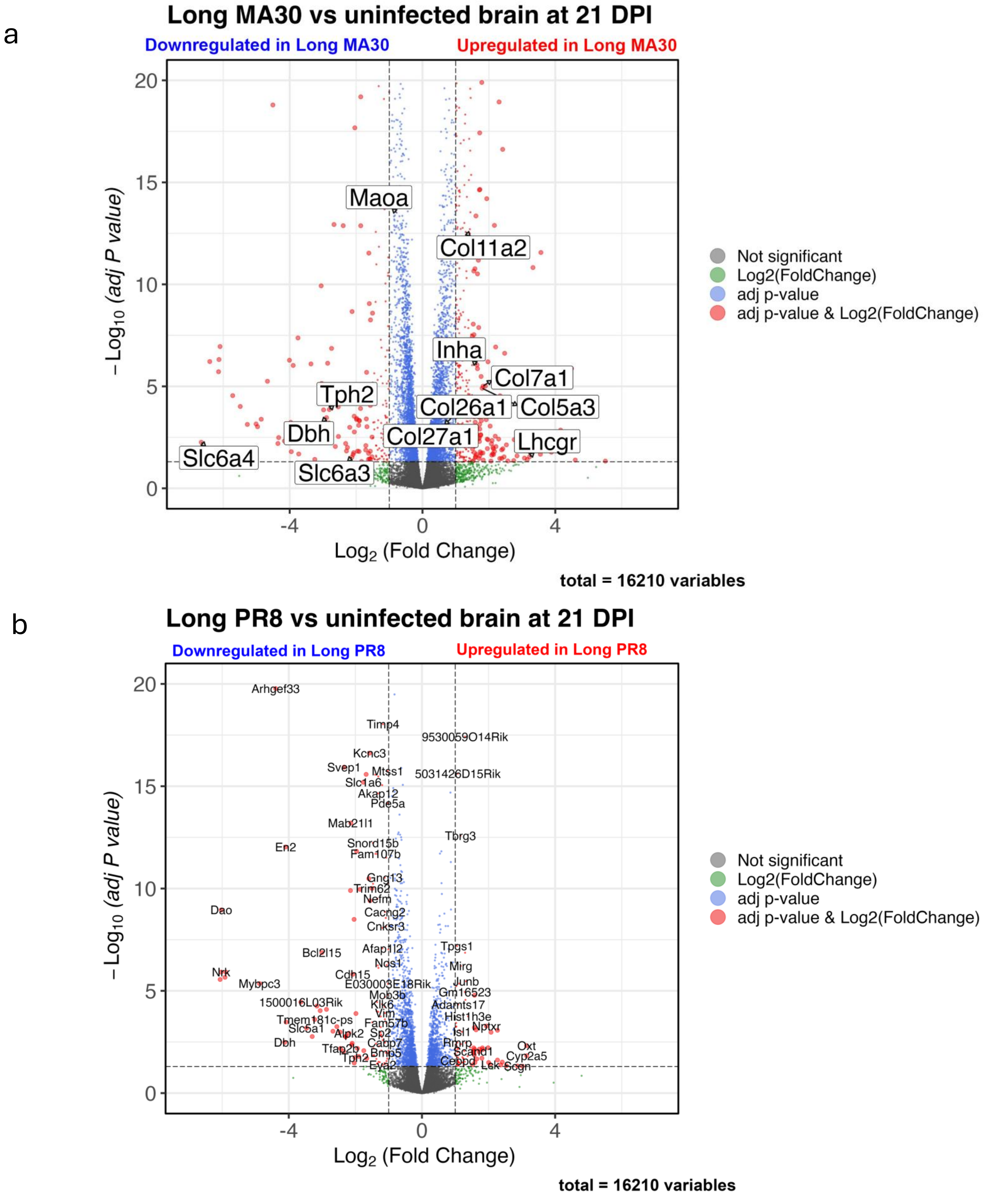

Supplementary Figure 18: Volcano plots of differentially expressed genes in MA30-infected and PR8-infected brain at 21 DPI reveal widespread transcriptional changes in MA30 but not PR8 versus uninfected controls.

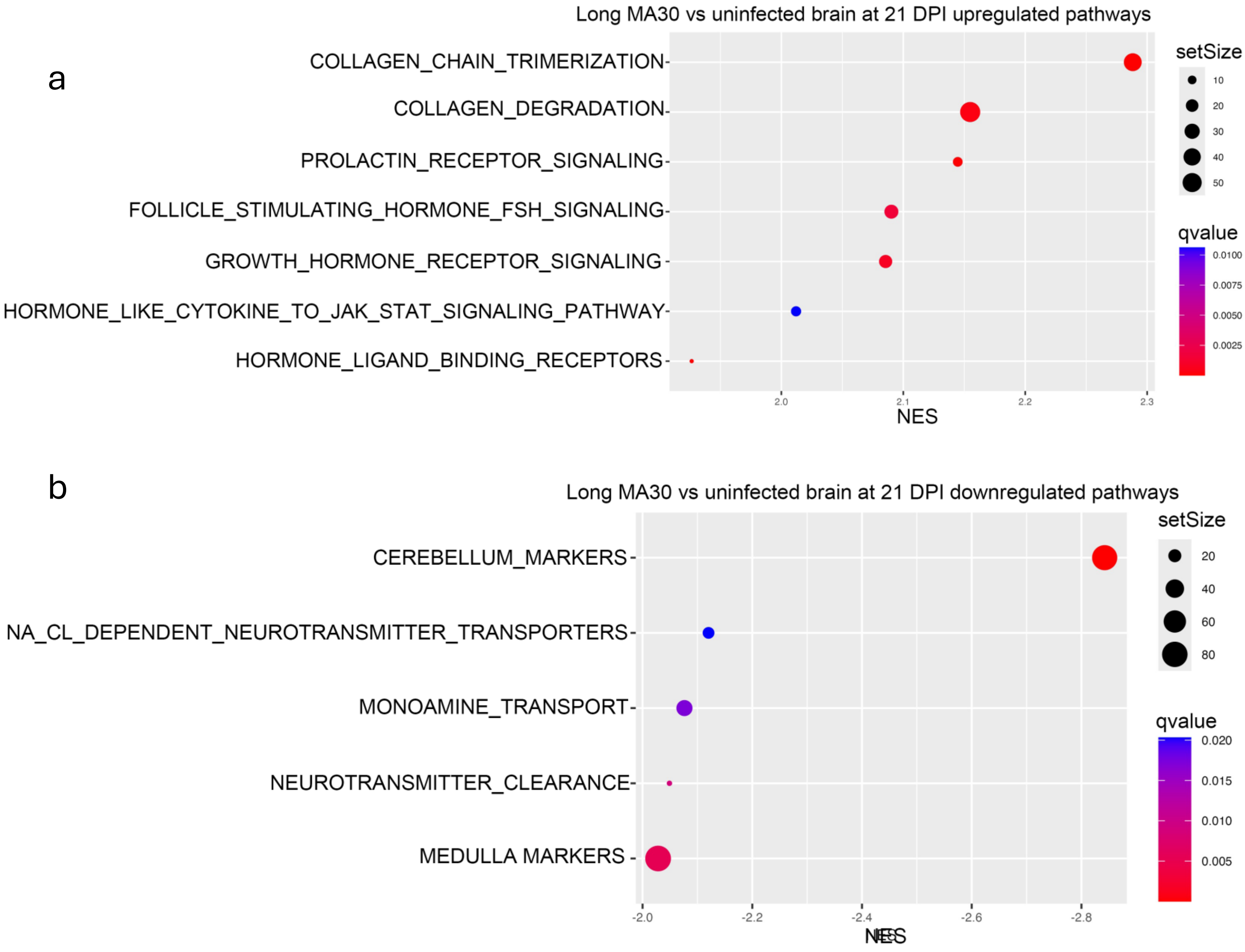

- **Supplementary Figure 19:** Pathway analysis of MA30-infected brains at 21 DPI shows **a)** upregulation of collagen degradation, hormone signaling, and cytokine-cytokine receptor interaction pathways, and **b)** downregulation of neurotransmitter clearance and synthesis signaling pathways.

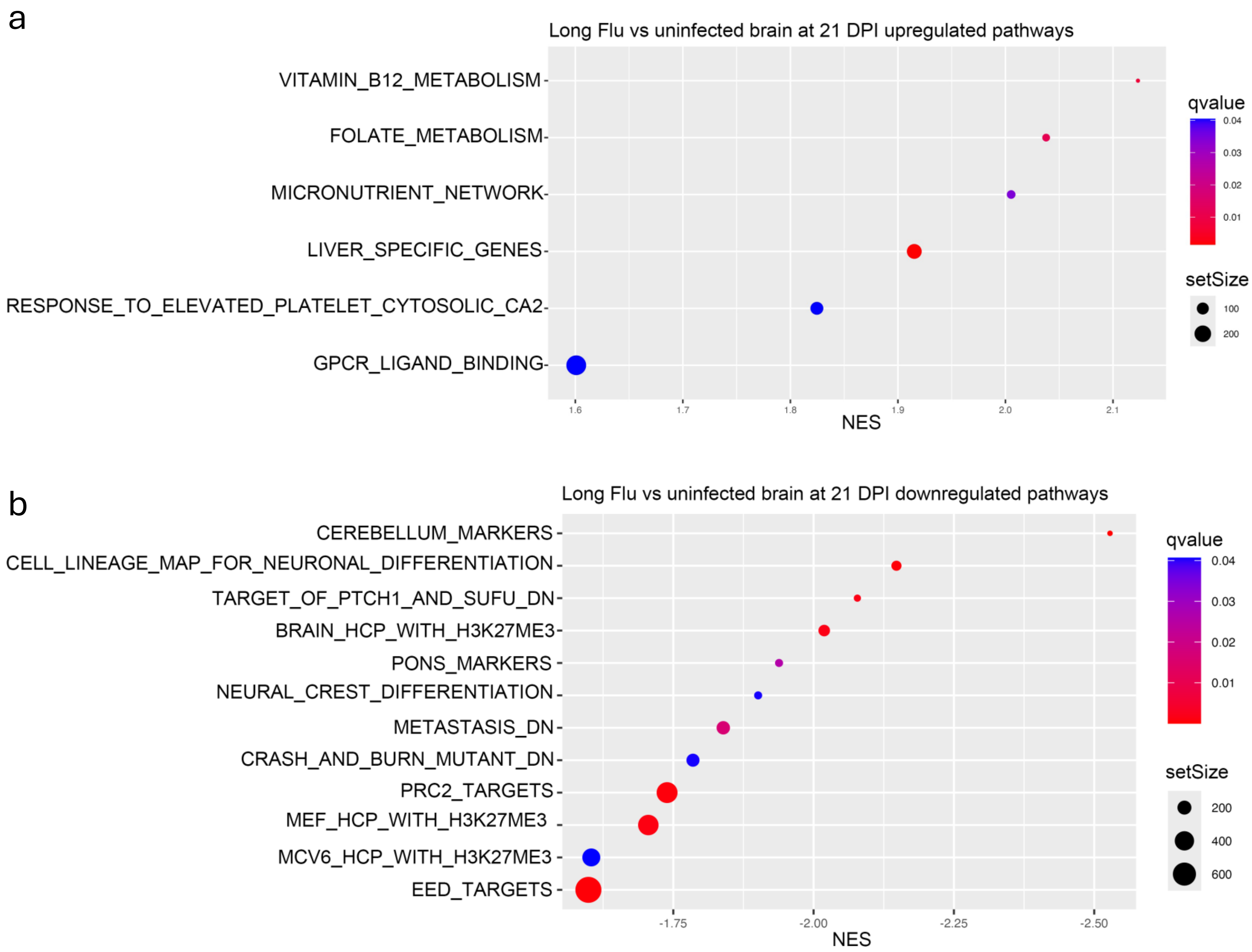

- **Supplementary Figure 20:** Pathway analysis of PR8-infected brains at 21 DPI reveals minimal long-term changes compared to uninfected controls.
