# Supplementary material for: Characterization of subchronic lung and brain consequences caused by mouse-adapted SARS-CoV-2 and influenza A infection of C57BL6 mice
Source: Front Immunol. 2026 Feb 25;17:1755141. doi: 10.3389/fimmu.2026.1755141 (PMC12975924; doi:10.3389/fimmu.2026.1755141)
Supplement: Supplementary file 2 [file DataSheet2.pdf]

Supplementary Tables

Supplementary Table 1: Upregulated complement genes and Cxcl10 in the lung of MA30 infected mice (21DPI) as compared to lung of uninfected mice.

gene_name	log2FoldChange	pvalue	padj
C3ar1	1.394647562	0.00099201	0.07152691
Cxcl10	1.962323487	0.00168161	0.0984048
C6	0.651665165	0.00840987	0.21837275
C1qb	1.465129956	0.01093966	0.2436436
C1qc	1.279534101	0.02590817	0.34099982
C1qa	1.113477051	0.05255841	0.44200874

pvalue and Padj indicated unadjusted nominal p value and adjusted p-value (Benjamini–Hochberg method) by DEseq2 respectively.

Supplementary Table 2: Upregulated coagulation factors and related genes in the lung of MA30 infected mice (21DPI) as compared to lung of PR8 infected mice (21DPI).

gene_name	log2FoldChange	pvalue	padj
Serpina1b	4.533200396	0.00506756	0.05834076
Serpina1c	4.396282204	0.01337967	0.10583669
Serpina3k	6.064357522	0.03153257	0.17470231

pvalue and Padj indicated unadjusted nominal p value and adjusted p-value (Benjamini–Hochberg method) by DEseq2 respectively.

Supplementary Table 3: upregulated coagulation factors and Fibriogen family genes, and downregulated complement genes in the lung of MA30 infected mice (5-7DPI) as compared to lung of PR8 infected mice (8-9DPI).

gene_name	log2FoldChange	pvalue	padj
Serpina3k	5.43931455	0.00017156	0.00112425
C2	-0.5940616	0.00087797	0.00439232
Serpina9	2.72004758	0.00165691	0.00750205
C4a	-1.2063384	0.01105501	0.03522914
Fgl1	2.28952652	0.02254878	0.06233798
Alb	6.39982169	5.1612E-06	5.6641E-05
Cfb	-0.5801602	3.9423E-05	0.00032013
C5ar2	-0.7381695	0.00072273	0.00374146

pvalue and Padj indicated unadjusted nominal p value and adjusted p-value (Benjamini–Hochberg method) by DEseq2 respectively.

Supplementary Table 4: Upregulated hormone genes, and downregulated neurotransmitter synthesis and transport related genes in the brain of MA30 infected mice (5-7DPI) as compared to brain of uninfected mice

gene_symbol	log2FoldChange	pvalue	padj
Prl	7.652978245	1.55E-06	
Gh	7.544486981	2.38E-06	0.006315
Cfd	8.65284614	2.67E-08	
Maoa	-0.303100356	0.006034	0.004773

pvalue and Padj indicated unadjusted nominal p value and adjusted p-value (Benjamini–Hochberg method) by DEseq2 respectively.

Supplementary Table 5: Downregulated hormone genes in the brain of MA30 infected mice (5-7 DPI) as compared to brain of PR8 infected mice (8-9 DPI)

gene_name	log2FoldChange	pvalue	padj
Gh	-3.0416264	0.08038809	0.19673807
Pomc	-3.8146771	0.00031268	
Prl	-3.9233989	0.00015479	

pvalue and Padj indicated unadjusted nominal p value and adjusted p-value (Benjamini–Hochberg method) by DEseq2 respectively.

Supplementary Table 6: Upregulated hormone genes in the brain of MA30 infected mice (21 DPI) as compared to brain of uninfected mice

gene_symbol	Long_COVID_Brain_19vsUninfected_Control_Brain_fold_change	pvalue	padj
Pomc	4.089114371	0.000158172	
Prl	10.47442385	1.60E-11	
Gh	12.23549185	7.34E-15	

pvalue and Padj indicated unadjusted nominal p value and adjusted p-value (Benjamini–Hochberg method) by DEseq2 respectively.

Supplementary Table 7: Upregulated hormone-related genes, ECM remodeling genes, and IL6 signaling genes in the brain of MA30 infected mice (21 DPI) as compared to brain of PR8 infected mice (21 DPI)

gene_name	log2FoldChange	pvalue	padj
Col16a1	2.70969171	2.8297E-29	1.6461E-26
Mmp17	0.43779859	7.5433E-07	8.8527E-06
Stat3	0.4362853	9.44E-06	8.1815E-05
Mmp24	0.42028565	0.00023777	0.00134654
Cebpb	1.1509528	0.01896894	0.05531689
Fshb	4.43570599	0.19131591	
Gh	7.443726	3.45E-10	
Pomc	3.64328754	0.00066141	
Prl	4.74041717	1.15E-05	

pvalue and Padj indicated unadjusted nominal p value and adjusted p-value (Benjamini–Hochberg method) by DEseq2 respectively.
